# Determining the critical factors of air-conditioning innovation using an integrated model of fuzzy Kano-QFD during the COVID-19 pandemic: The perspective of air purification

**DOI:** 10.1371/journal.pone.0255051

**Published:** 2021-07-27

**Authors:** Yinyun Yu, Congdong Li, Weiming Yang, Wei Xu

**Affiliations:** 1 School of Management, Jinan University, Guangzhou, China; 2 School of Management, Shenyang University of Technology, Shenyang, China; University of Defence in Belgrade, SERBIA

## Abstract

At present, people are demanding better indoor air quality during the COVID-19 pandemic. In addition to maintaining the basic functions, new air-conditioning should also add air purification functions to improve indoor air quality and reduce the possibility of virus transmission. Nowadays, there is lack of research results on the innovation of air-conditioning. The aim of this study is to present a two-stage mathematical model for identifying critical manufacturing factors in the innovation process of air conditioning. In this paper, Kano and quality function deployment (QFD) are used to analyze the critical factors affecting air-conditioning innovation. Some studies have proposed using Kano-QFD model to analyze product innovation, but the study only studies one stage, which loses the analysis of the subsequent stages of product innovation. Based on this, this paper studies the priority method of two-stage critical factors for air-conditioning innovation. Firstly, the questionnaire survey and fuzzy sets are used to collect demand information of multi-agent (customers and professional technicians). Secondly, the Kano model is used to classify and calculate satisfaction of multi-agent. Then, QFD is used to transform multi-agent demands into engineering property indexes (first stage) and technical property indexes (second stage) and calculate the weight of each index. Finally, the applicability and superiority of this method is illustrated by taking the central air-conditioning as an example.

## 1. Introduction

COVID-19 can rapidly and massively spread through the air. We need to purify the air in order to reduce the virus in the air. Air-conditioning with air purification function become the innovation orientation of air-conditioning. New air-conditioning not only need to increase the air purification function, but also should improve the existing basic functions. Air-conditioning innovation is a kind of incremental innovation of existing products. Product innovation is systematic engineering with complex manufacturing processes, high investment costs, high technical requirements, and high R&D risks [1]. The identification of critical factors for air-conditioning innovation is at the beginning of the product life cycle. Scientifically identifying the market’s demand for products can effectively extend the product life cycle [2]. Under the conditions of cost, manufacturing technology and process equipment, choosing the critical factors that can best cater to customer demand preferences as the priority development factors can help companies gain market competitiveness and best corporate performance.

Safizadeh pointed out that the company’s choice of different product design methods will make the company have different competitive advantages. When the company’s product innovation does not match customer needs, the company’s performance will suffer [3]. Efficient and accurate selection of the critical factors in the product design process can make the company invincible in the fiercely competitive market [4]. Wang and Zhou pointed out that only product innovation that fully meets the demands of customers can be finally accepted by the market, so the research on demand is very important [5].

Scholars research on product innovation from a large number of perspectives. Eum et al. [6] connect production and innovation show that production advantages play an important role in technological innovation and product innovation. Dangelico et al. [7] study green product innovation based on the dynamic capability perspective of sustainable development. The current research subjects mostly focus on other product innovation, there is no research focusing on air-conditioning innovation. In addition, The innovation of product design only considers customer demands from the market, not technological innovation. Furthermore, the researchers ignore ambiguity and uncertainty of customer’s demands due to the limitation of customer’s personal knowledge background. These problems have become practical problems faced by enterprises in product innovation. In order to fill this gap, aiming at the identification of critical factors in the process of air-conditioning innovation, this paper proposes a method for identifying critical factors of air-conditioning innovation that considers customer demands and technological innovation in a fuzzy environment.

There are great differences and uncertainties between customers’ and experts’ demands for product innovation. Fuzzy sets can handled effectively numerical and linguistic uncertainties, which can transform uncertain information into quantifiable fuzzy number [8, 9]. According to the driving factors of product innovation (demand-driven and technology-driven), collect product demands from the market customers and product designers through questionnaire surveys. The fuzzy sets are used to transform the demand information into fuzzy value to minimize the deviation of product demand information. Kano model is widely used in the research of demand classification and prioritization, which can obtain the nonlinear relationship between product performance and customer satisfaction [10]. QFD provides a robust framework to translate the customer demands into engineering or technical characteristics [11, 12]. It can provide valuable information about which functions need to be improved and which functions should be replaced. Therefore, this paper chooses Kano and QFD methods to build the model, which transforms product requirements of multi-agent to manufacturing features, so as to identify the key critical factors that have the greatest impact on the manufacturing process.

In this study, we want to develop a (product planning and process planning stage) two-stage model of critical factors for manufacturing process and give a fusion method of fuzzy number and Kano-QFD. Then, this model is applied to the innovation process of air conditioning. The research questions of the paper are the following:

- How can the opinions of multi-agent (customers and professional technicians) for product be better integrated into product innovation?
- How to integrate fuzzy number into Kano model in requirements research?
- How to use QFD to give a (product planning and process planning stage) two-stage model of critical factors to make the theoretical model closer to the actual manufacturing process?

The main contributions of this study include three points. Firstly, the traditional product innovation only considers the customer’s demand for product function, but product innovation comes from not only market demand, but also technological innovation and upgrading in practice, so we consider the influence of many factors (demand-driven factors and technology-driven factors). Secondly, fuzzy sets are integrated into Kano model to reduce the deviation between demand function and manufacturing characteristics. Thirdly, this paper uses QFD Model to identify the critical factors in the two stages of the product manufacturing process, and adopts two-stage analysis, which is closer to the real manufacturing situation. Specifically, the proposed new method is comprised by the following phases:

- In the process of air-conditioning innovation, we consider two aspects of innovation driving factors: demand driven and technology driven, that is, market customers’ demands for air-conditioning and designers’ demands for air-conditioning.
- Due to the differences of knowledge background and demand expression between multi-agent, we introduce fuzzy sets to collect the demand of multiple agents, so that the demand is closer to the actual market situation
- The Kano model is used to classify the demands of multi-agent and calculate the weights of different demands.
- The QFD model is used to decompose customer demands into engineering property indexes (product planning stage) and technical property indexes (process planning stage). After the decomposition of these two stages, customer demands can be effectively transformed into production tasks of design department and production department.
- The priority of air-conditioning innovation indexes under multi-agent demand preferences is calculated based on Kano-QFD.

The remainder of this paper is organized as follows. After reviewing some relevant literature in Section 2, we describe the research problem in Section 3 and give a method in Section 4. In Section 5, we provide a case study about air-conditioning innovation. Section 6 concludes this work.

## 2. Literature reviews

The selection of key quality characteristics (KQCs) that are significantly associated with product quality is essential for improving product quality [13]. Wiiam et al. [14] believe that in addition to technical factors, market factors also play an important role in product innovation. Li et al. [15] proposes a key quality characteristics (KQCs) selection method, which want to get maximizing feature (i.e., quality characteristic) importance and minimizing percentage of selected features. After studying the sample of 2126 manufacturing companies, Liao et al. [16] find that customer demands have a positive moderating effect on the impact of innovation intensity and innovation ability. Choudhary and Singh [17] took the hotel industry as the research object and discusses the impact of customer demand and competitiveness on propensity for innovation in the hospitality sector. Customers want flexibility so that they can choose specific products and services according to their needs [18]. Considering the uncertainty of manufacturing resources, Xu and Yu [19] proposed a discrete manufacturing decision-making model under fuzzy environment, which comprehensively considered customer demand preference and supplier profit maximization. Dragan et al. [20] introduce fuzzy numbers into Best-Worst Method (BWM) and todim (Iterative Multi-Criteria Decision Making) methods, and presents a multi criteria prioritization methodology for automobile industry. Torkayesh et al. [21] propose a new MCDM (multi-criteria decision-making) method, the stratified MCDM, this method can effectively deal with the uncertainty of environment and the fluctuation of index weight. Further more, he uses geographic information system (GIS), best and worst method (BWM) and compromise method (MARCOS) to rank the landfill location. This method can obtain a decision matrix with the ideal and anti ideal under grey interval set considering sustainability factors [22]. Yazdani et al. [23] study the problem of supplier evaluation and propose a interval valued fuzzy neutrosophic (IVFN) model. Taking into account the uncertainty of expert evaluation information, he adopt linguistic measures and their corresponding neutrosophic values to obtain this information. Tirkolaee et al. [24] use the fuzzy analysis method (FANP), the fuzzy decision-making trial and evaluation laboratory (DEMATEL) and the technique for order preference by similarity to an Ideal solution (TOPSIS) to rank and select suppliers. The practitioners can better express their opinions (their direction and intensity) based on the fuzzy technique.

Inaccurate identification of market demands will result in poor matching between product innovation and market demands. Kano model is a method to study the relationship between product quality and customer satisfaction [25]. QFD model is a method to transform customer demands into product design or innovation [26]. Jia et al. [27] use the Kano model for mobile phone software development. He uses the Kano model to identify the customer demands for software and determine the priority of software development modules. Based on the differences of decision makers, Yang et al. [28] propose an improved Kano model of customer demand preferences to determine the priority of customer demands. Loucanova and Olsiakova [29] apply the Kano model to the innovation process of wood products. The results show that consumers have a positive understanding of product development. Zhang et al. [30] study customer satisfaction demand identification methods and proposed a simple and easy fuzzy group decision-making method. Take the innovative design of kitchenware as an example to verify the applicability of the method. Silva et al. [31] described a method that integrates Quality Function Deployment with Theory of Inventive Problem Solving, which requires technical innovation specified from an analysis of customers’ needs. Ocampo et al. [32] give a models of fuzzy QFD multiple attribute decision making (QFD-MADM), which helps to promote sustainability by incorporating requirements at an early stage of design process. Taking food processing as the research object, the innovation stage of food processing in the Philippines was studied. Chen et al. [33] evaluate the relationship between customer requirements (CRs) and design requirements (DRs) and the correlations among DRs in QFD processes based on the QFD. This study adopts experimental design and fuzzy set to collect the data. On this basis, the paper constructed a fuzzy mathematical model of each CR s satisfaction level, which can represent the interaction between the CR s satisfaction level and the fulfillment levels of DRs. Cho et al. [34] give a new mode, considering user’s personal preferences for requirements, which combines the benefits of QFD with those of TOPSIS. The model can be used to analyze positive/negative ideal criteria and limit values between multiple market products and user requirements.

According to Table 1, we can obtain:

1. The existing studies focus on the analysis of innovation factors and product innovation design. Researchers prefer to adopt the method of model research and there are abundant research results on the deterministic environment.
2. The analysis of innovation factors is an important research in product innovation. However, nearly all related papers in the domain of product innovation have considered the problem from the single perspective (customer’s viewpoint), while in the real situation, the product innovation is influenced customer demands and technological progresses. In this study, the influencing factors of product innovation are analyzed from two aspects: market driven and technology driven.
3. Most studies focus on product innovation design, proving that product design is a key point in product innovation, so the paper focuses on the selection of critical factors of air-conditioning innovation. However, the majority of the studies limit themselves to product planning while losing control over other subsequent phases of process planning. Thus, this paper attempts to give a two-stage model based on Kano-QFD.
4. Most studies are carried out in a deterministic environment, but the uncertain environment is more in line with the real situation. The paper focus on product innovation in uncertain environment. In the process of this study, the fuzzy number is used to deal with the uncertainty problems in the real situation, which improves the applicability of the model.
5. Due to the availability and uncertainty of information in decision making, the fuzziness of human emotion and recognition, it is often difficult to accurately evaluate and convey the emotion and recognition of decision making objects. An expert more efficiently employs their implicit knowledge, experiences, and information through language evaluation. A fuzzy set is a versatile tool both for linguistic and numerical modeling, which can transform linguistic information into corresponding computable fuzzy numbers, while grey interval numbers, hierarchical theory and neutral sets cannot deal with this problem. Therefore, when dealing with language problems, the fuzzy set is adopted.

**Table 1:** Product innovation literature (partial) analysis.

Literature	Topics			Method		Scenarios		Problems
	Innovation factors	Innovation design	Innovation quality	Theoretical analysis	Model method	Certainty scenario	Uncertainty scenario	
[14]	√			√		√		How market factors affect product innovation
[15]	√				√	√		The impact of key factors on quality of product innovation
[16]	√			√		√		The influence of customer demand on innovation
[17]	√				√	√		The impact of customer demand and competitiveness on propensity for innovation
[19]	√				√		√	The influence of customer demand preference on product manufacturing
[20]	√				√		√	Key factors in automobile manufacturing
[25]			√		√	√		The relationship between product quality and customer satisfaction
[26]		√			√	√		Analyzing and dealing with the distortions in customer requirements transmission process of QFD
[27]		√						The priority of software development modules
[28]	√				√	√		Prioritizing customer demands
[29]		√			√	√		The influence of customer demand on product innovation
[30]		√			√		√	The innovative design of kitchenware in fuzzy environment
[31]		√			√	√		Technological innovation based on customer demands
[32]		√			√		√	Integrated multiphase sustainable product design with a QFD-MADM) framework
[33]		√			√		√	The relationship between CRs and DRs and the correlations among DRs in QFD processes.
[34]		√			√	√		The influence of user’s personal preference on product innovation

## 3. Problem description

The paper considers the air-conditioning innovation design for multi-agent demand preferences in fuzzy environment. Air-conditioning innovation from two aspects: demand-driven and technology-driven. Customs may design products according to their own demands. However, the product demands will differ due to customs particular and distinct preferences [35, 36]. At the same time, product designers will improve the product in conjunction with the development of technology. In addition, product innovation may also be affected by objective conditions such as technological constraints and environmental constraints. Only by accurately identifying the market’s demands for product innovation, can maximize the market’s acceptance and adoption of products, and ultimately achieve the desired innovation benefits [37]. Along with the study of Ding et al., our paper takes all possible individual preferences among the indexes into account [38].

**Fig 1:**
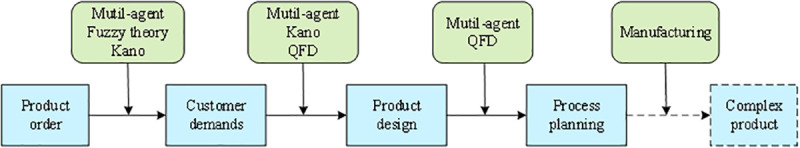
The logic diagram.

Through market surveys, we can get air-conditioning demand information of multi-agent. Using fuzzy sets to transform demand information into corresponding fuzzy values. According to the demand information and the Kano model, product demands are divided into five categories: Must-be, One-dimensional, Attractive, Indifferent, Reverse. At the same time, the Kano model is used to calculate the satisfactory of demands. The QFD two-stage model is used to transform customer demand into engineering property indexes and technical property indexes respectively, and calculate the weight of each index considering multi-agent demand preferences. Thus, the indexes ranking are obtained and the key product innovation indexes which have the greatest impact on demand are determined. The logic of paper is shown in Fig 1.

Considering the situation where there are three entities, i.e., market customer group (MC), process design group (DT), product manufacturing group (MT), that give theirs demands and satisfactory. Let $CR^{S_{d}}=\{CR_{1}^{S_{d}},\ldots ,CR_{n}^{S_{d}}\}$, *i* = 1,…,*n* represent product demands of three entities; $S^{S_{d}}=\{S_{1}^{S_{d}},\ldots ,S_{n}^{S_{d}}\}$ represent product customer satisfaction of three entities. Therefore, the importance degree of the *j*-th engineering property index (*EW*_*j*_) can be calculated by Eq (1).


$$EW_{j}=\sum_{sd}\sum_{i=1}^{n}S_{i}^{S_{d}}•CW_{ij}^{S_{d}},\;j=1,\ldots ,m,\;S_{d}\in \{MC_{d1},DT_{d2},MT_{d3}|d1\in R,d2\in R,d3\in R\}$$ (1)

In which $CW_{ij}^{S_{d}}$ represents the correlation matrix between product satisfactory and engineering property indexes given by multi-agent.

Based on this, We can further obtain the importance degree of technical property indexes as

$$TW_{k}=\sum_{z_{d}}\sum_{j=1}^{m}EW_{j}^{Z_{d}}•CT_{jk}^{Z_{d}}k=1,\ldots ,l,\;Z_{d}\in \{DT_{d2},MT_{d3}|d2\in R,d3\in R\}$$ (2)

Here, *TW*_*k*_ represents the importance degree of the *k*-th technical property index, $CT_{jk}^{Z_{d}}$ represents the correlation matrix between engineering property indexes and technical property indexes given by multi-agent

An intuitive practice is to determine accurate numbers of different dimensions with respect to each indicator, and then employ typical aggregation function to get importance degrees of indicators [39]. However, it is often difficult for customers to express their specific demands for products with precise values. For example, customers want food to be fresher. Liao [40, 41] point out that the use of fuzzy sets to represent an expert’s preferences when assessing a linguistic variable, increases the flexibility of eliciting and representing linguistic information. Based on fuzzy sets and multi-agent demand preferences, this paper integrates the Kano model and the QFD model to rank the importance orders of the key product innovation factors for a air-conditioning.

## 4. Methodology

In this section, a priority methodology is proposed for dealing with air-conditioning innovation, the main feature of which is considering multi-agent demand preferences and vague expressions.

**Fig 2:**
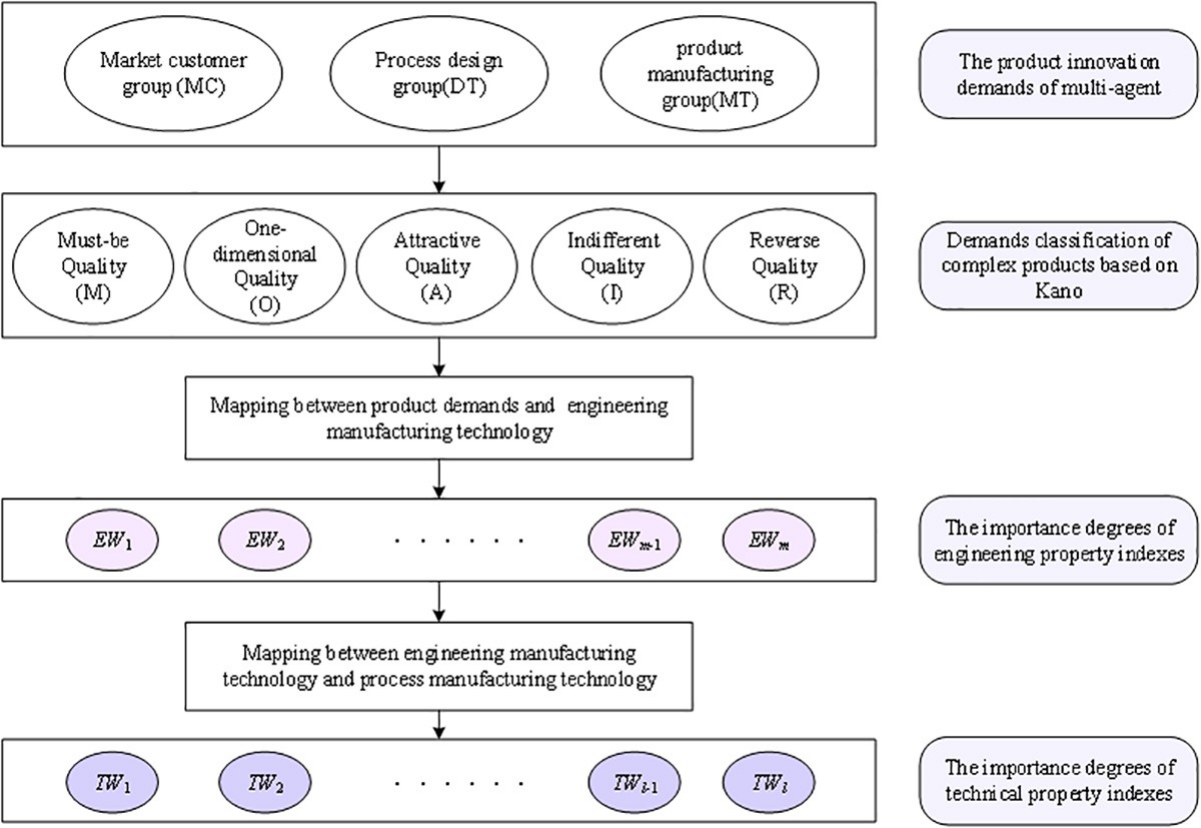
Methodology.

Our methodology can be divided into three-fold, as shown in Fig 2. Firstly, the multi-agent demand preference information of air-conditionings is collected, and then the demand preference information is transformed into fuzzy value based on fuzzy sets. Secondly, we can obtain the classify of product demands and the demand weights of multi-dimensional satisfactory according to multi-agent demand preferences. Further more, according to the demand analysis principle of the QFD model, the product demands are mapped to engineering design and process design. Finally, the weights of engineering property indexes and technical property indexes are obtained and sorted.

### 4.1 Calculation of multi-agent satisfaction based on Kano model

#### 4.1.1 Kano analysis of multi-agent demands

**Fig 3:**
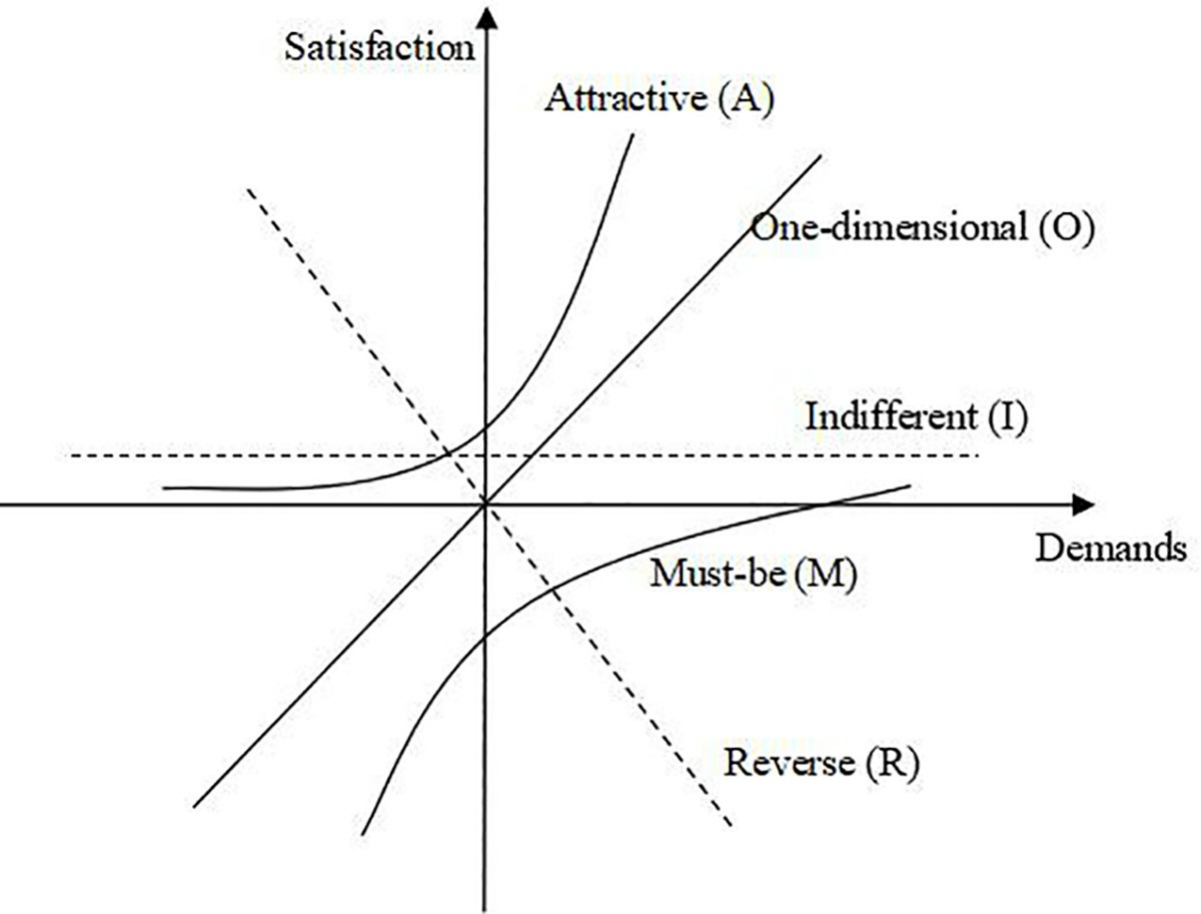
Kano model.

Kano model is a mathematical model for classifying and prioritizing customer demands proposed by quality management expert Kano N. [42]. Based on the analysis of customer demands, the model divides customer demands into Must-be (M), One-dimensional (O), Attractive (A), Indifferent (I), Reverse (R), as shown in Fig 3.

The Kano model is used to identify multi-agent demands in the innovative design of air-conditioning. On the one hand, it can divide multi-agent demands scientifically and reasonably. On the other hand, it can help the product design department to effectively know and control multi-agent demands for products.

In the Kano questionnaire, each demand is designed into two dimensions: “With” and “Without”. Under each dimension, there are five types of answers: “Favorite”, “Necessary”, “Indifferent”, “Reluctant” and “Disgusting”. According to the two-dimensional attributes, the multi-agent demands are classified, so as to realize demand classification of air-conditioning. The corresponding demand classification judgment matrix is shown in Table 2. Let *CR* = {*CR*_1_,…,*CR*_*n*_} represents multi-agent demands, in which *CR*_*i*_ represents demand of *i*-th agent. Here multi-agent includes market customer group (MC), process design group (DT), product manufacturing group (MT).

**Table 2:** Demand classification judgment matrix.

		With				
		Favorite	Necessary	Indifferent	Reluctant	Disgusting
Without	Favorite	-	A	A	A	O
	Necessary	R	I	I	I	M
	Indifferent	R	I	I	I	M
	Reluctant	R	I	I	I	M
	Disgusting	R	R	R	R	-

#### 4.1.2 Satisfaction function

If a demand is an indifferent demand, no matter whether the company increases or decreases the demand, multi-agent satisfactory and dissatisfaction will not change. Therefore, this paper does not analyze indifferent demand satisfactory calculation.

*4*.*1*.*2*.*1 Attractive demand satisfaction function*. Attractive demand refers to the unexpected demand of customers. If air-conditioning have this function or feature, customer satisfaction can increase rapidly; If a air-conditioning does not have this function or feature, customer satisfaction will not decrease. If the *i*-th demand is attractive demand, its satisfactory can be calculate by following

$$S_{i}=a_{i}^{a}e^{\left(x_{i}^{*}\right)}+b_{i}^{a}\;i=1,\ldots ,n$$ (3)

Here,

$$a_{i}^{a}=\frac{CS_{i}-DS_{i}}{e-1},\;b_{i}^{a}=\frac{CS_{i}-eDS_{i}}{e-1}$$ (4)

in which, *S* = {*S*_1_,…,*S*_*n*_} represents a set of multi-agent satisfactory. *S*_*i*_ represents satisfactory of multi-agent for the *i*-th demand (*CR*_*i*_). $a_{i}^{a}$ and $b_{i}^{a}$ are the adjustment coefficient of satisfaction function. satisfactory and dissatisfaction of *CR*_*i*_ represented by *CS*_*i*_ and *DS*_*i*_ respectively [43], which can can be calculated by Eqs (5) and (6).


$$CS_{i}=\frac{M_{i}+O_{i}+A_{i}}{M_{i}+O_{i}+A_{i}+I_{i}+R_{i}}$$ (5)

$$DS_{i}=\frac{I_{i}+R_{i}}{M_{i}+O_{i}+A_{i}+I_{i}+R_{i}}$$ (6)

Here, *M*_*i*_ represents the number of people who think the demand is the product must-be, and then, *O*_*i*_, *A*_*i*_, *I*_*i*_ and *R*_*i*_ also represent the number of people.

$x_{i}^{*}$ is the *i*-th agent demand expectation after normalization [44].


$$x_{i}^{*}=\left\{ \begin{array}{c} 1,x_{i}\geq x_{i}^{Ae}\\ \frac{x_{i}-x_{i}^{Ie}}{x_{i}^{Ae}-x_{i}^{Ie}},x_{i}^{Ae}>x_{i}\geq x_{i}^{Ie}\\ 0,x_{i}^{Ie}>x_{i} \end{array}\right.$$ (7)

Where, *x*_*i*_ is the actual evaluation value of multi-agent for *CR*_*i*_; $x_{i}^{Ie}$ is minimum expectation; $x_{i}^{Ae}$ is maximum expectation.

*4*.*1*.*2*.*2 One-dimensional demand satisfaction function*. One-dimensional demand refers to the functions and features that customers want air-conditioning to possess. The higher realization degree of one-dimensional demand, the greater customer satisfaction. There is a positive correlation between realization degree and customer satisfaction. If the *i*-th demand is one-dimensional demand, its satisfactory can be calculate by following

$$S_{i}=a_{i}^{o}x_{i}^{*}+b_{i}^{o}$$ (8)

$a_{i}^{o}$ and $b_{i}^{o}$ are adjustment coefficient of one-dimensional demand satisfaction function, which can be calculated by Eq (9).


$$a_{i}^{o}=CS_{i}-DS_{i},\;b_{i}^{o}=DS_{i}$$ (9)

*4*.*1*.*2*.*3 Must-be demand satisfaction function*. Must-be demand refer to the functions and features that customers think air-conditioning should possess. If air-conditioning has this function or feature, customer satisfaction will not be significantly increased. However, if air-conditioning do not have this function or feature, customer satisfaction will be significantly reduced. If the *i*-th demand is must-be demand, its satisfactory can be calculate by following

$$S_{i}=a_{i}^{m}(-e^{-x_{i}^{∴}})+b_{i}^{m}$$ (10)

$a_{i}^{m}$ and $b_{i}^{m}$ are adjustment coefficient of must-be demand satisfaction function, which can be calculated by Eq (11).


$$a_{i}^{m}=\frac{e(CS_{i}-DS_{i})}{e-1},\;b_{i}^{m}=\frac{eCS_{i}-DS_{i}}{e-1}$$ (11)

*4*.*1*.*2*.*4 Reverse demand satisfaction function*. Reverse demand refers to the functions and features that customers do not want air-conditioning to have. If air-conditioning has this function or feature, customer satisfaction will decrease. The greater degree of realization, the greater dissatisfaction. There is a negative correlation between reverse demand and customer satisfaction. If the *i*-th demand is reverse demand, its satisfaction can be calculate by following

$$S_{i}=a_{i}^{r}x_{i}^{*}+b_{i}^{r}$$ (12)

$a_{i}^{r}$ and $b_{i}^{r}$ are adjustment coefficient of reverse demand satisfaction function, which can be calculated by Eq (13)。

$$a_{i}^{r}=-(CS_{i}-DS_{i}),\;b_{i}^{r}=DS_{i}$$ (13)

Where, demand expectation $x_{i}^{∴}$ can be calculated by Eq (14).


$$x_{i}^{∴}=\left\{ \begin{array}{c} 0,x_{i}\geq x_{i}^{Ae}\\ \frac{x_{i}-x_{i}^{Ie}}{x_{i}^{Ae}-x_{i}^{Ie}},x_{i}^{Ae}>x_{i}\geq x_{i}^{Ie}\\ 1,x_{i}^{Ie}>x_{i} \end{array}\right.$$ (14)

#### 4.1.3 Modification of satisfaction function

In order to make the calculated customer satisfaction closer to the actual situation, we need to modify satisfaction function. Tan et al. [45] propose a method to modify satisfaction function.


$$SW_{i}=S_{i}\times AI_{i}^{*}$$ (15)

Here, $AI_{i}^{*}$ is adjustment coefficient, which can be calculated by Eqs (16 and 17).


$$AI_{i}^{*}={\left(AI_{i}\right)}^{\frac{1}{k}}$$ (16)

$$AI_{i}=\frac{x_{i}}{x_{i}^{Ae}}$$ (17)

Here, *k* is the Kano factor; *AI*_*i*_ is the initial adjustment coefficient of satisfaction function.

Based on this, we can get four types of satisfaction functions for Must-be demand, One-dimensional demand, Reverse demand and Attractive demand, as shown in Table 3.

**Table 3:** Satisfaction function.

KC	*a*_*i*_	*b*_*i*_	*S*_*i*_	*SW*_*i*_
A	$\frac{CS_{i}-DS_{i}}{e-1}$	$\frac{CS_{i}-eDS_{i}}{e-1}$	$\frac{CS_{i}-DS_{i}}{e-1}e^{x_{i}^{*}}+\frac{CS_{i}-eDS_{i}}{e-1}$	$\left(\frac{CS_{i}-DS_{i}}{e-1}e^{x_{i}^{*}}+\frac{CS_{i}-eDS_{i}}{e-1}\right)•{\left(\frac{x_{i}}{x_{i}^{Ae}}\right)}^{\frac{1}{k}}$
O	*CS*_*i*_−*DS*_*i*_	*DS*_*i*_	$(CS_{i}-DS_{i})x_{i}^{*}+DS_{i}$	$((CS_{i}-DS_{i})x_{i}^{*}+DS_{i})•{\left(\frac{x_{i}}{x_{i}^{Ae}}\right)}^{\frac{1}{k}}$
M	$\frac{e(CS_{i}-DS_{i})}{e-1}$	$\frac{eCS_{i}-DS_{i}}{e-1}$	$\frac{e(CS_{i}-DS_{i})}{e-1}(-e^{-x_{i}^{*}})+\frac{eCS_{i}-DS_{i}}{e-1}$	$\left(\frac{e(CS_{i}-DS_{i})}{e-1}(-e^{-x_{i}^{*}})+\frac{eCS_{i}-DS_{i}}{e-1}\right)•{\left(\frac{x_{i}}{x_{i}^{Ae}}\right)}^{\frac{1}{k}}$
R	−(*CS*_*i*_−*DS*_*i*_)	*DS*_*i*_	$-(CS_{i}-DS_{i})x_{i}^{*}+DS_{i}$	$(-(CS_{i}-DS_{i})x_{i}^{*}+DS_{i})•{\left(\frac{x_{i}}{x_{i}^{Ae}}\right)}^{\frac{1}{k}}$

### 4.2 Ranking model of critical factors

#### 4.2.1 QFD and fuzzy sets

**Fig 4:**
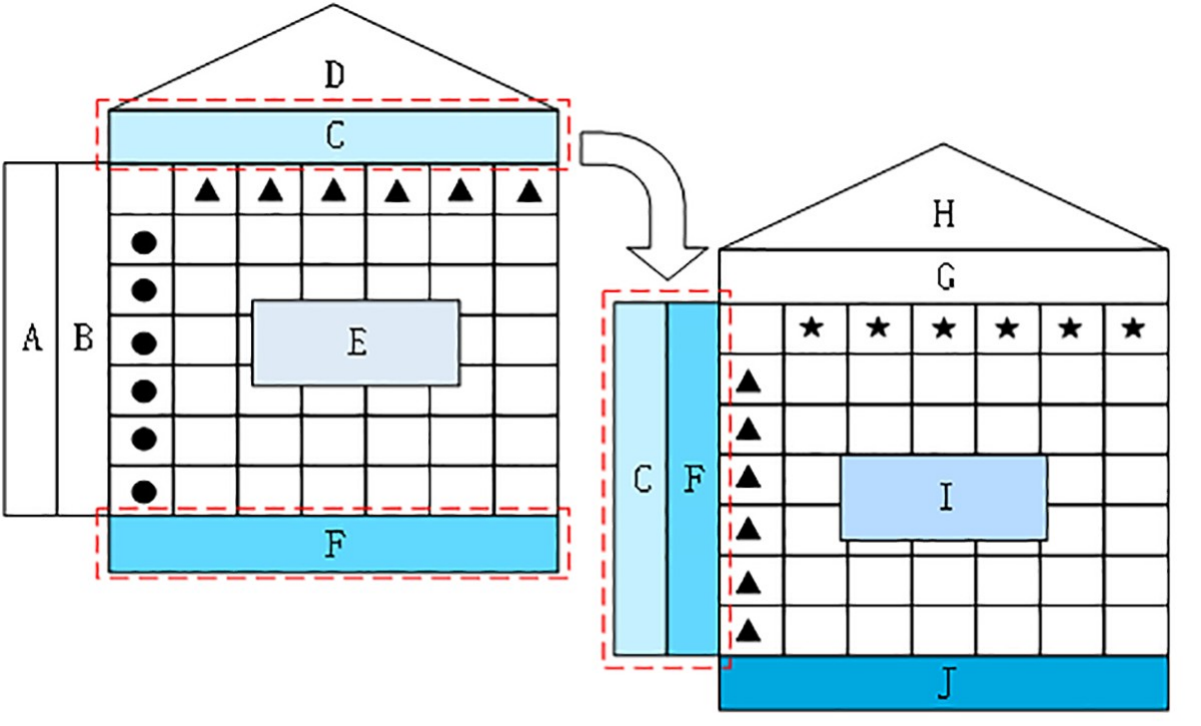
QFD quality model of two-stage. A: Multi-agent demand indexes. B: Satisfaction matrix of multi-agent demands. C: Engineering property indexes. D: Autocorrelation matrix of engineering property indexes. E: Correlation matrix between engineering property indexes and multi-agent demand indexes. F: Importance matrix of engineering property indexes. G: Technical property indexes. H: Autocorrelation matrix of technical property indexes. I: Correlation matrix between engineering property indexes and technical property indexes. J: Importance matrix of technical property indexes.

*4*.*2*.*1*.*1 QFD*. The most important function of QFD method is to transform customer demands into product manufacturing performances and determine the critical factors in the product manufacturing process [46]. QFD method is widely used in the product design stage to accurately understand customer demands for products. The QFD method uses a series of product planning matrices, the house of quality, to decompose customer demands in four stages, which are: product planning stage, process planning stage, part configuration stage, and production planning stage. Because this paper only identifies and analyzes the critical factors in the manufacturing process of air-conditioning, this paper only studies the two stages of product planning stage and process planning stage, as shown in Fig 4.

*4*.*2*.*1*.*2 Fuzzy sets*. Some product demands are difficult to quantify. At this time, using language descriptions is more in line with customers’ psychology of product functional requirements. Language description is more in line with customers’ psychological demands for product functions. For example, when customers evaluate food, language such as “fresh” and “not fresh” can better express customer satisfaction. Because of the uncertainty and fuzziness of language description, we adopt fuzzy sets to deal with it. The fuzzy sets proposed by Professor Zadeh [47] has proved to be an important tool for effectively dealing with the problems of ambiguity and uncertainty. This paper uses triangular fuzzy numbers to describe multi-agent demands.

Assuming that *B* is a fuzzy subset of fuzzy set *U*. For any *x* (*x*∈*U*), there is a corresponding *u*(*x*), *u*(*x*)∈[0,1]. we can say *u*(*x*) is membership function of *x*, that is fuzzy number. If *B* is a triangular fuzzy number, *B* = (*b*^1^,*b*^2^,*b*^3^), its membership function can be calculated by Eq (18).


$$u_{b}=\left\{ \begin{array}{c} 0,x\leq b^{1}\\ \frac{x-b^{1}}{b^{2}-b^{1}},b^{1}<x\leq b^{2}\\ \frac{b^{3}-x}{b^{3}-b^{2}},b^{2}<x\leq b^{3}\\ 0,b^{2}<x \end{array}\right.$$ (18)

#### 4.2.2 Fuzzy ranking model

*EP* = {*EP*_*1*_,*…*,*EP*_*m*_} represents a set of engineering property indexes, *EP*_*j*_ is the *j*-th engineering property index, *j* = 1,…,*m*. *TP* = {*TP*_1_,…,*TP*_*l*_} represents a set of technical property indexes, *TP*_*k*_ is the *k*-th technical property index, *k* = 1,…,*l*. According to the *n* multi-agent demands for air-conditioning, the designer gives the corresponding *m* engineering property indexes and *l* technical property indexes.

Due to the complex manufacturing process of air-conditioning, in addition to multi-agent demands, design feasibility and process feasibility should also be considered for innovative design of products. Therefore, the product demands in this paper is not only customer demands of market, but also design demands and manufacturing demands. In order to facilitate readers’ better reading, this paper summarizes the three agents of Market customer group (MC), process design group (DT), product manufacturing group (MT) into an expert team. Due to the difference of knowledge background and requirement understanding of expert teams, the design of air-conditioning is fuzzy and ambiguous. This paper uses fuzzy sets to evaluate the two-stage QFD model. The corresponding fuzzy evaluation values are shown in Table 4.

**Table 4:** Triangular fuzzy number of fuzzy evaluation.

Relevance	Symbol	Triangular fuzzy number
Extremely relevant	●	(0.8,0.9,1.0)
Strong relevant	◎	(0.6,0.7,0.8)
Middle relevant	○	(0.4,0.5,0.6)
Weak relevant	△	(0.2,0.3,0.4)
irrelevant	▲	(0,0.1,0.2)

*4*.*2*.*2*.*1 Engineering property indexes ranking*. Let $D_{jg}^{Z_{d}}$ represents the autocorrelation between engineering property indexes *EP*_*j*_ and *EP*_*g*_ given by expert *Z*_*d*_,*j*≠*g*∈[1,*m*]. Let $E_{ij}^{Z_{d}}$ represents the correlation between multi-agent demand *CR*_*i*_ and engineering property index *EP*_*j*_ given by expert *Z_d_*, *i*∈[1, *n*], $Z_{d}\in \{MC_{d1},DT_{d2},MT_{d3}|d1\in R,d2\in R,d3\in R\}.\;D_{jg}^{Z_{d}}$ and $E_{ij}^{Z_{d}}$ are triangular fuzzy numbers. According to the triangular fuzzy number calculation rules, the average value of $D_{jg}^{Z_{d}}$ and $E_{ij}^{Z_{d}}$ given by *r* experts is calculated.


$$E_{ij}^{Z}=\frac{\sum_{d=1}^{r}E_{ij}^{Z_{d}}}{r},\;D_{jg}^{Z}=\frac{\sum_{d=1}^{r}D_{jg}^{Z_{d}}}{r}\;r\in (h_{1},h_{2},h_{3})$$ (19)

Here, *h*_1_ represents the numbers of *MC*; *h*_2_ represents the numbers of *DT*; *h*_3_ represents the numbers of *MT*.

The average expert evaluation information can be obtained by Eq (19). Furthermore, we can obtain the correlation matrix (CM) of multi-agent demands and engineering property indexes, the autocorrelation matrix (AM) of engineering property indexes, as shown below.


$$CM={\left[E_{ij}^{Z}\right]}_{\left(n\times m\right)},\;AM={\left[D_{jg}^{Z}\right]}_{\left(m\times m\right)}$$ (20)

$$CW=CM•AM={\left[E_{ij}^{Z}•D_{jg}^{Z}\right]}_{n\times m}={\left[CW_{ij}\right]}_{n\times m}$$ (21)

*CW* is the improved correlation between multi-agent demands and engineering property indexes.

According to Eqs (15) and (21), we can get satisfaction function and correlation matrix. Eq (22) is used to obtain the *j*-th project performance indexes and normalize it.


$$EW_{j}=\sum_{i=1}^{n}SW_{i}•CW_{ij}$$ (22)

$$EW_{j}^{*}=\frac{EW_{j}}{\sum_{j=1}^{m}EW_{j}}$$ (23)

According to Eq (23), we can get the normalized importance set of engineering property indexes, $EW^{*}=\{EW_{1}^{*},\ldots ,EW_{m}^{*}\}$.

*4*.*2*.*2*.*2 Technical property indexes ranking*. Let $I_{jk}^{Z_{d}}$ represents the correlation between engineering property index *EP*_*j*_ and technical property index *TP*_*k*_ given by expert *Z*_*d*_,*j*∈[1,*m*], *k*∈[1,*l*]. Let $H_{kp}^{Z_{d}}$ represents the autocorrelation between technical property indexes *TP*_*k*_ and *TP*_*p*_ given by expert *Z*_*d*_, *p*∈[1,*l*], *Z*_*d*_∈{*DT*_*d*2_, *MT*_*d*3_|*d*2∈*R*, *d*3∈*R*}.

According to the calculation rule of Eqs (19)–(21), we can get the improved correlation (*CT*)between engineering property index *EP*_*j*_ and technical property index *TP*_*k*_, as follow.


$$CT={\left[I_{jp}^{Z}•H_{pk}^{Z}\right]}_{m\times l}={\left[CT_{jk}\right]}_{m\times l}$$ (24)

Multiply the importance of the engineering property indexes obtained by Eq (23) and the relationship matrix obtained by Eq (24) to obtain the importance of technical property indexes, as shown in Eq (25).


$$TW_{k}=\sum_{j=1}^{m}EW_{j}^{*}•CT_{jk}$$ (25)

$$TW_{k}^{*}=\frac{TW_{k}}{\sum_{k=1}^{l}TW_{k}}$$ (26)

According to Eq (26), we can get the normalized importance set of technical property indexes, $TW^{*}=\{TW_{1}^{*},\ldots ,TW_{l}^{*}\}$.

#### 4.2.3 Ranking model

According to the previous paper, we can get the importance set of engineering property indexes and the importance set of technical property indexes, EW* and TW*. According to the triangular fuzzy number calculation rule, EW* and TW* are still triangular fuzzy numbers, EW* = (EW*^1^, EW*^2^, EW*^3^) and TW* = (TW*^1^, TW*^2^, TW*^3^). Since fuzzy numbers cannot be compared numerically, we need to convert fuzzy numbers into exact numbers. The comparison and sorting of fuzzy numbers requires the introduction of a cut-set (that is the confidence level), which is an important method to turn fuzzy numbers into exact numbers [48]. The fuzzy number converted into an exact number under the *α* cut-set, which is calculated by Eqs (27) and (28).


$${\left(\bar{E}{\bar{W}}^{*}\right)}_{\alpha }=[(EW^{*2}-EW^{*3})\alpha +EW^{*1},EW^{*3}-(EW^{*3}-EW^{*2})\alpha ]$$ (27)

$${\left(\bar{T}{\bar{W}}^{*}\right)}_{\alpha }=[(TW^{*2}-TW^{*3})\alpha +TW^{*1},TW^{*3}-(TW^{*3}-TW^{*2})\alpha ]$$ (28)

${\left(\bar{E}{\bar{W}}^{*}\right)}_{\alpha }^{L}$ and ${\left(\bar{E}{\bar{W}}^{*}\right)}_{\alpha }^{U}$ respectively represent the upper and lower lines of the fuzzy number EW* under the *α* cut-set.

In order to effectively describe the ambiguity and uncertainty of the air-conditioning design process, a weighted modified average level *α* cut-set defuzzification method is adopted. This method can effectively solve the problem of difficulty in sorting caused by the aggregation of multi-index triangular fuzzy numbers. The calculation Eq is (29).


$$QE_{j}=\frac{1}{N}\sum_{i=1}^{N}\alpha_{i}•\left(\frac{{\left(\bar{E}{\bar{W}}^{*}\right)}_{\alpha_{1}}^{L}+{\left(\bar{E}{\bar{W}}^{*}\right)}_{\alpha_{1}}^{U}}{2}\right)$$ (29)

Here, *QE*_*j*_ is the important of the *j*-th engineering property index. *α* = {*α*_*i*_|*α*_1_,…,*α*_*N*_} is a set of *α*, 0≤*α*_*i*_≤1, *i* = 1,…,*N*.

Similarly, we can use Eq (30) to calculate the importance of each technical property index, and sort them, so as to identify the critical factors in the process of air-conditioning innovation design.


$$QT_{k}=\frac{1}{N}\sum_{i=1}^{N}\alpha_{i}•\left(\frac{{\left(\bar{T}{\bar{W}}^{*}\right)}_{\alpha_{1}}^{L}+{\left(\bar{T}{\bar{W}}^{*}\right)}_{\alpha_{1}}^{U}}{2}\right)$$ (30)

*QT*_*k*_ is the important of the *k*-th technical property index.

## 5. Example simulation

**Fig 5:**
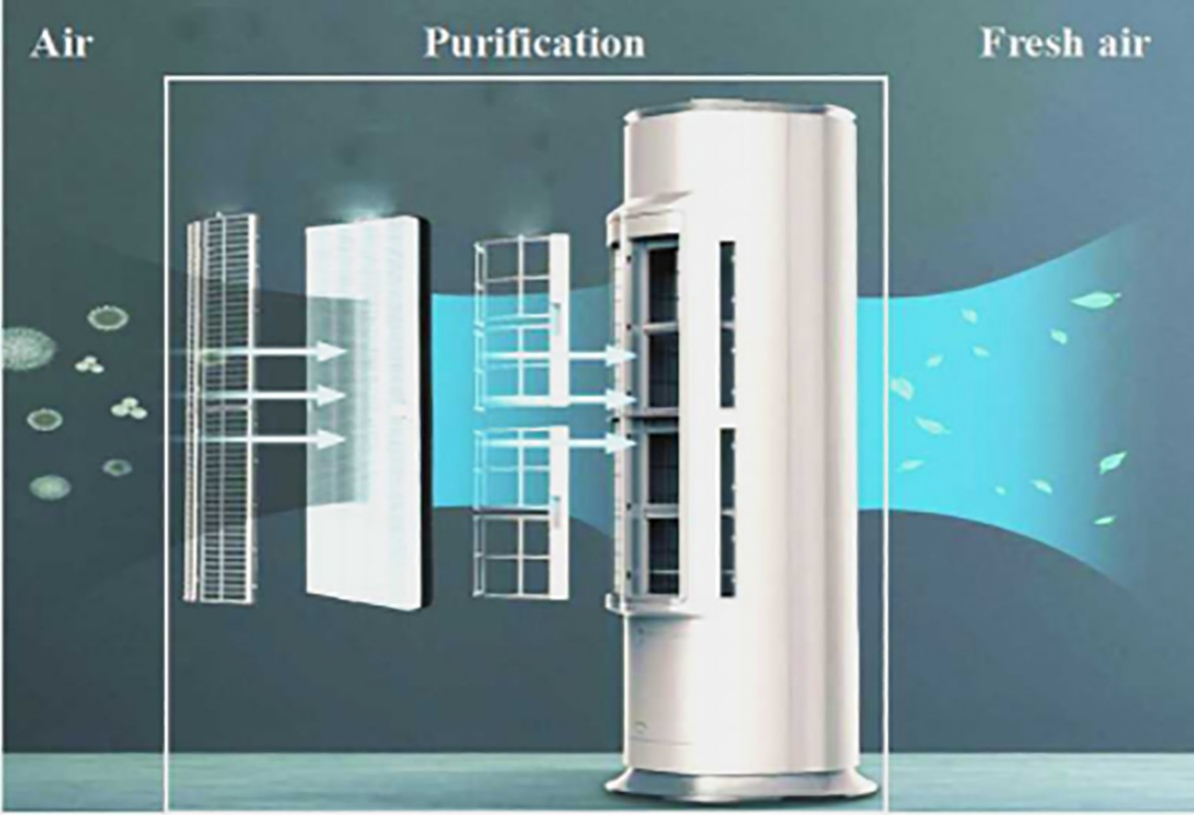
Product structure.

In this paper, the KFR air-conditioning of Gree Electric Appliances, Inc.of Zhuhai (that is simply as Gree) is taken as the research object. The product structure is shown in Fig 5. The KFR air-conditioning will be put on the market in 2018. Now it is planned to transform or replace part of its function and utility in order to improve air purification capacity during the COVID-19 pandemic.

The company organized 5 experts from the process design department to carry out demands and design concept, according to product order. Four customer demands, eight engineering property indexes and fifteen technical property indexes were obtained, as shown in Table 5. According to the characteristics of product demands, Kano questionnaire was designed and collected online. A total of 200 questionnaires were collected, including market customer group demands (MC), process design group demands (DT), product manufacturing group demands (MT), with weight ratios of 0.5, 0.3 and 0.2. After removing the invalid data, the classification of product demands and the average value of evaluation information are obtained, as shown in Table 6.

**Table 5:** Innovative design indicators of air-conditioning.

Demand indexes		Engineering property indexes		Technical property indexes	
Stability	*CR*_1_	Motor units	*EP*_1_	Air inlet/outlet design	*TP*_1_
Reliability	*CR*_2_	Intelligent protection	*EP*_2_	Data storage design	*TP*_2_
Efficiency	*CR*_3_	Monitoring settings	*EP*_3_	Condition monitoring design	*TP*_3_
Environment-friendly	*CR*_4_	Operation panel	*EP*_4_	Energy adjustment range design	*TP*_4_
		Operating efficiency	*EP*_5_	Operating range design	*TP*_5_
		Low-density diffuser	*EP*_6_	Motor protection design	*TP*_6_
		Control settings	*EP*_7_	Bearing protection design	*TP*_7_
		Operating ranges	*EP*_8_	Component over-temperature protection design	*TP*_8_
				Low\high voltage protection design	*TP*_9_
				Group control module technology	*TP*_10_
				Touch screen design	*TP*_11_
				Filter screen design	*TP*_12_
				Oil-free design	*TP*_13_
				Refrigerant application design	*TP*_14_

**Table 6:** Demand classification and evaluation information.

	Demand indexes	M	O	A	I	R	Total	KC	*x*_*i*_	$x_{i}^{Ie}$	$x_{i}^{Ae}$
MC	*CR*_1_	45	33	22	14	36	150	M	0.008	0.192	0.452
	*CR*_2_	24	30	56	15	25	150	A	0.898	0.359	0.981
	*CR*_3_	10	34	48	35	23	150	A	0.164	0.157	0.350
	*CR*_4_	16	52	22	35	25	150	O	0.324	0.201	0.475
DT	*CR*_1_	17	2	2	4	5	30	M	0.050	0.263	0.505
	*CR*_2_	5	3	13	6	3	30	A	0.351	0.382	0.678
	*CR*_3_	14	5	2	4	5	30	M	0.205	0.214	0.517
	*CR*_4_	11	1	9	2	7	30	M	0.259	0.252	0.765
MT	*CR*_1_	8	2	1	4	5	20	M	0.275	0.377	0.764
	*CR*_2_	11	2	0	4	3	20	M	0.302	0.069	0.998
	*CR*_3_	8	4	3	2	3	20	M	0.143	0.199	0.965
	*CR*_4_	7	5	2	4	2	20	M	0.368	0.124	0.325

Combining Eqs (4)–(21) to obtain the weight of the importance of multi-agent demands, as shown in Table 7.

**Table 7:** The importance of multi-agent demands.

	Demand indexes	$x_{i}^{*}$	*CS*	*DS*	*a*_*i*_	*b*_*i*_	*S*_*i*_	*SW*_*i*_
MC	*CR*_1_	0.035	0.667	0.333	0.527	0.861	0.315	0.048
	*CR*_2_	0.867	0.733	0.267	0.272	0.005	0.651	0.288
	*CR*_3_	0.036	0.613	0.387	0.132	-0.255	-0.118	-0.006
	*CR*_4_	0.449	0.600	0.400	0.200	0.400	0.490	0.463
DT	*CR*_1_	0.050	0.700	0.300	0.633	0.933	0.268	0.053
	*CR*_2_	0.078	0.700	0.300	0.233	-0.067	0.184	0.011
	*CR*_3_	0.300	0.700	0.300	0.633	0.933	0.078	0.091
	*CR*_4_	0.0136	0.700	0.300	0.633	0.933	0.291	0.010
MT	*CR*_1_	-0.264	0.550	0.450	0.158	0.608	0.487	-0.336
	*CR*_2_	0.251	0.650	0.350	0.475	0.825	0.215	0.108
	*CR*_3_	0.010	0.750	0.250	0.791	1.041	0.242	0.005
	*CR*_4_	1	0.700	0.300	0.633	0.933	-0.787	-4.845

According to the importance symbols shown in Table 3, the correlation evaluation information of QFD in two stages given by experts is collected, as shown in Tables 8 and 9. In the first stage, the expert groups include market customer group (MC), process design group (DT), product manufacturing group (MT). Considering the limitation of market customer group knowledge background, in the second stage, the expert groups only include process design group (DT), product manufacturing group (MT).

**Table 8:** The evaluation information in the first stage (One export).

	*EP*_1_	*EP*_2_	*EP*_3_	*EP*_4_	*EP*_5_	*EP*_6_	*EP*_7_	*EP*_8_
*EP*_1_	●	◎	▲	▲	●	◎	◎	●
*EP*_2_	◎	●	●	▲	◎	◎	○	▲
*EP*_3_	▲	●	●	●	▲	▲	◎	▲
*EP*_4_	▲	▲	●	●	○	▲	●	▲
*EP*_5_	●	◎	▲	○	●	●	△	◎
*EP*_6_	◎	◎	▲	▲	●	●	▲	△
*EP*_7_	◎	○	◎	●	△	▲	●	▲
*EP*_8_	●	▲	▲	▲	◎	△	▲	●
*CR*_1_	●	○	●	▲	△	◎	◎	●
*CR*_2_	◎	●	○	▲	◎	●	◎	◎
*CR*_3_	●	▲	△	●	●	◎	●	◎
*CR*_4_	●	▲	▲	▲	○	▲	▲	▲

**Table 9:** The evaluation information in the second stage (One export).

	*TP*_1_	*TP*_2_	*TP*_3_	*TP*_4_	*TP*_5_	*TP*_6_	*TP*_7_	*TP*_8_	*TP*_9_	*TP*_10_	*TP*_11_	*TP*_12_	*TP*_13_	*TP*_14_
*TP*_1_	●	▲	◎	●	◎	◎	△	△	△	▲	▲	○	○	△
*TP*_2_	▲	●	△	▲	▲	▲	△	▲	▲	○	△	▲	▲	▲
*TP*_3_	◎	△	●	●	●	◎	◎	◎	◎	▲	△	○	▲	▲
*TP*_4_	●	▲	●	●	●	●	○	○	○	▲	▲	◎	◎	▲
*TP*_5_	◎	▲	●	●	●	●	▲	◎	◎	▲	▲	◎	▲	▲
*TP*_6_	◎	▲	◎	●	●	●	▲	●	●	▲	▲	○	▲	▲
*TP*_7_	△	△	◎	○	▲	▲	●	○	○	▲	▲	◎	▲	▲
*TP*_8_	△	▲	◎	○	◎	●	○	●	●	▲	▲	◎	▲	▲
*TP*_9_	△	▲	◎	○	◎	●	○	●	●	▲	▲	▲	◎	▲
*TP*_10_	▲	○	▲	▲	▲	▲	▲	▲	▲	●	◎	▲	▲	▲
*TP*_11_	▲	△	△	▲	▲	▲	▲	▲	▲	◎	●	▲	▲	▲
*TP*_12_	○	▲	○	◎	◎	○	◎	◎	◎	▲	▲	●	◎	◎
*TP*_13_	○	▲	▲	◎	▲	▲	▲	▲	▲	▲	▲	◎	●	◎
*TP*_14_	△	▲	▲	▲	▲	▲	▲	▲	▲	▲	▲	◎	◎	●
*EP*_1_	●	●	●	●	●	△	△	△	△	▲	▲	○	○	○
*EP*_2_	◎	○	○	△	△	●	●	●	●	▲	◎	▲	▲	▲
*EP*_3_	▲	○	○	▲	○	○	○	○	◎	●	▲	▲	▲	▲
*EP*_4_	▲	▲	▲	▲	▲	▲	▲	▲	▲	●	●	▲	▲	▲
*EP*_5_	◎	▲	▲	◎	○	○	○	○	○	▲	▲	●	●	◎
*EP*_6_	△	▲	▲	◎	△	▲	▲	△	△	▲	▲	◎	△	●
*EP*_7_	▲	▲	▲	▲	○	○	○	○	○	▲	◎	▲	▲	◎
*EP*_8_	●	▲	▲	●	○	○	○	○	○	▲	▲	○	◎	▲

According to fuzzy sets, the evaluation information given by experts is transformed into corresponding fuzzy numbers. Eq (19) is used to obtain the fuzzy mean value of the correlation matrix and the autocorrelation matrix for multi-agent. In the first stage, the fuzzy matrix of the correlation matrix (CM) and the autocorrelation matrix (AM) are shown in Table 10.

**Table 10:** Fuzzy value of evaluation information in the first stage (DT).

	*EP*_1_	*EP*_2_	*EP*_3_	*EP*_4_	*EP*_5_	*EP*_6_	*EP*_7_
*EP*_1_	(1.000,1.000,1.000)	(0.514,0.602,0.690)	(0.033,0.131,0.209)	(0.066,0.164,0.242)	(0.754,0.835,0.924)	(0.522,0.610,0.698)	(0.483,0.571,0.659)
*EP*_2_	(0.514,0.602,0.690)	(1.000,1.000,1.000)	(0.718,0.799,0.888)	(0.035,0.133,0.211)	(0.582,0.670,0.758)	(0.438,0.526,0.614)	(0.372,0.482,0.592)
*EP*_3_	(0.033,0.131,0.209)	(0.718,0.799,0.888)	(1.000,1.000,1.000)	(0.691,0.772,0.861)	(0.024,0.122,0.200)	(0.057,0.155,0.233)	(0.417,0.505,0.593)
*EP*_4_	(0.066,0.164,0.242)	(0.035,0.133,0.211)	(0.691,0.772,0.861)	(1.000,1.000,1.000)	(0.409,0.519,0.629)	(0.051,0.149,0.227)	(0.765,0.846,0.935)
*EP*_5_	(0.754,0.835,0.924)	(0.582,0.670,0.758)	(0.024,0.122,0.200)	(0.409,0.519,0.629)	(1.000,1.000,1.000)	(0.755,0.836,0.925)	(0.133,0.217,0.301)
*EP*_6_	(0.522,0.610,0.698)	(0.438,0.526,0.614)	(0.057,0.155,0.233)	(0.051,0.149,0.227)	(0.755,0.836,0.925)	(1.000,1.000,1.000)	(0.150,0.248,0.326)
*EP*_7_	(0.483,0.571,0.659)	(0.372,0.482,0.592)	(0.417,0.505,0.593)	(0.765,0.846,0.935)	(0.133,0.217,0.301)	(0.150,0.248,0.326)	(1.000,1.000,1.000)
*EP*_8_	(0.775,0.856,0.945)	(0.135,0.233,0.311)	(0.076,0.174,0.252)	(0.038,0.136,0.214)	(0.468,0.556,0.644)	(0.111,0.195,0.279)	(0.034,0.132,0.210)
*CR*_1_	(0.804,0.885,0.974)	(0.582,0.670,0.762)	(0.512,0.593,0.682)	(0.053,0.151,0.229)	(0.216,0.300,0.384)	(0.492,0.580,0.668)	(0.638,0.727,0.815)
*CR*_2_	(0.689,0.777,0.865)	(0.582,0.670,0.763)	(0.515,0.625,0.735)	(0.094,0.192,0.270)	(0.543,0.631,0.719)	(0.752,0.833,0.922)	(0.532,0.620,0.708)
*CR*_3_	(0.794,0.875,0.964)	(0.582,0.670,0.764)	(0.185,0.269,0.353)	(0.755,0.836,0.925)	(0.792,0.873,0.962)	(0.494,0.582,0.670)	(0.792,0.873,0.962)
*CR*_4_	(0.773,0.854,0.943)	(0.582,0.670,0.765)	(0.088,0.186,0.264)	(0.120,0.218,0.296)	(0.342,0.452,0.562)	(0.046,0.144,0.222)	(0.054,0.152,0.230)

According to Table 10, the fuzzy evaluation information values (CM and AM) given by the DT expert group are obtained. Eq (21) is used to obtain the improved correlation CM* between multi-agent demands *CR* and engineering property indexes *EP*. And then, Eq (22) is used to obtain the absolute importance of engineering property indexes *EW*. Furthermore, we obtained the normalized importance of EW* by Eq (23). Due to the same calculation process and limited paper layout, the paper only gives the absolute importance and the normalized importance of market customer group (MC) and product manufacturing group (MT), as shown in Table 10, the specific calculation steps are not described in this paper.

According to the Eq (27) and Table 11, the fuzzy number is transformed into the exact number, which the results are shown in Table 12. Eq (29) is used to weighted average the interval number. We can get the importance of engineering property indexes and sort them by Eq (29), and the importance mean of each index is calculated by mean method. which shown in Table 13.

**Table 11:** The importance of engineering property indexes.

	*EW*							
	*EP*_1_	*EP*_2_	*EP*_3_	*EP*_4_	*EP*_5_	*EP*_6_	*EP*_7_	*EP*_8_
*MC*	(0.513,0.671,0.850)	(0.410,0.574,0.758)	(0.255,0.406,0.566)	(0.281,0.434,0.601)	(0.479,0.641,0.828)	(0.368,0.511,0.673)	(0.352,0.503,0.672)	(0.333,0.468,0.620)
*DT*	(0.543,0.709,0.898)	(0.428,0.602,0.796)	(0.269,0.428,0.597)	(0.303,0.465,0.640)	(0.507,0.678,0.874)	(0.387,0.538,0.708)	(0.377,0.535,0.713)	(0.353,0.495,0.655)
*MT*	(0.453,0.596,0.758)	(0.358,0.507,0.674)	(0.219,0.355,0.500)	(0.244,0.383,0.534)	(0.423,0.570,0.739)	(0.323,0.453,0.600)	(0.308,0.445,0.598)	(0.296,0.418,0.556)
	EW*							
*MC*	(0.092,0.159,0.284)	(0.074,0.136,0.254)	(0.046,0.096,0.189)	(0.050,0.103,0.201)	(0.086,0.152,0.277)	(0.066,0.121,0.225)	(0.063,0.120,0.225)	(0.060,0.111,0.207)
*DT*	(0.092,0.159,0.284)	(0.073,0.135,0.251)	(0.046,0.096,0.189)	(0.052,0.104,0.202)	(0.086,0.152,0.276)	(0.066,0.121,0.224)	(0.064,0.120,0.225)	(0.060,0.111,0.207)
*MT*	(0.091,0.159,0.289)	(0.072,0.135,0.257)	(0.044,0.095,0.191)	(0.049,0.103,0.204)	(0.085,0.153,0.282)	(0.065,0.122,0.229)	(0.062,0.119,0.228)	(0.060,0.112,0.212)

**Table 12:** The importance under *α* cut-set (taking DT as an example).

*α*	${\left(\bar{E}{\bar{W}}^{*}\right)}_{\alpha }$	*EP*_1_	*EP*_2_	*EP*_3_	*EP*_4_	*EP*_5_	*EP*_6_	*EP*_7_	*EP*_8_
0	${\left(\bar{E}{\bar{W}}^{*}\right)}_{\alpha }^{L}$	0.850	0.758	0.566	0.601	0.828	0.673	0.672	0.620
	${\left(\bar{E}{\bar{W}}^{*}\right)}_{\alpha }^{U}$	0.513	0.410	0.255	0.281	0.479	0.368	0.352	0.333
0.1	${\left(\bar{E}{\bar{W}}^{*}\right)}_{\alpha }^{L}$	0.832	0.740	0.550	0.585	0.809	0.656	0.655	0.605
	${\left(\bar{E}{\bar{W}}^{*}\right)}_{\alpha }^{U}$	0.529	0.426	0.270	0.296	0.495	0.382	0.367	0.347
0.2	${\left(\bar{E}{\bar{W}}^{*}\right)}_{\alpha }^{L}$	0.814	0.721	0.534	0.568	0.790	0.640	0.638	0.590
	${\left(\bar{E}{\bar{W}}^{*}\right)}_{\alpha }^{U}$	0.545	0.443	0.285	0.311	0.511	0.396	0.382	0.360
0.3	${\left(\bar{E}{\bar{W}}^{*}\right)}_{\alpha }^{L}$	0.796	0.703	0.518	0.551	0.772	0.624	0.621	0.574
	${\left(\bar{E}{\bar{W}}^{*}\right)}_{\alpha }^{U}$	0.561	0.459	0.300	0.327	0.528	0.411	0.397	0.374
0.4	${\left(\bar{E}{\bar{W}}^{*}\right)}_{\alpha }^{L}$	0.779	0.685	0.502	0.535	0.753	0.608	0.604	0.559
	${\left(\bar{E}{\bar{W}}^{*}\right)}_{\alpha }^{U}$	0.576	0.475	0.315	0.342	0.544	0.425	0.412	0.387
0.5	${\left(\bar{E}{\bar{W}}^{*}\right)}_{\alpha }^{L}$	0.761	0.666	0.486	0.518	0.734	0.592	0.588	0.544
	${\left(\bar{E}{\bar{W}}^{*}\right)}_{\alpha }^{U}$	0.592	0.492	0.330	0.357	0.560	0.439	0.427	0.401
0.6	${\left(\bar{E}{\bar{W}}^{*}\right)}_{\alpha }^{L}$	0.743	0.648	0.470	0.501	0.716	0.576	0.571	0.529
	${\left(\bar{E}{\bar{W}}^{*}\right)}_{\alpha }^{U}$	0.860	0.762	0.568	0.607	0.836	0.677	0.679	0.626
0.7	${\left(\bar{E}{\bar{W}}^{*}\right)}_{\alpha }^{L}$	0.725	0.629	0.454	0.484	0.697	0.559	0.554	0.514
	${\left(\bar{E}{\bar{W}}^{*}\right)}_{\alpha }^{U}$	0.623	0.525	0.361	0.388	0.593	0.468	0.458	0.428
0.8	${\left(\bar{E}{\bar{W}}^{*}\right)}_{\alpha }^{L}$	0.707	0.611	0.438	0.468	0.678	0.543	0.537	0.498
	${\left(\bar{E}{\bar{W}}^{*}\right)}_{\alpha }^{U}$	0.639	0.541	0.376	0.404	0.609	0.482	0.473	0.441
0.9	${\left(\bar{E}{\bar{W}}^{*}\right)}_{\alpha }^{L}$	0.689	0.592	0.422	0.451	0.660	0.527	0.520	0.483
	${\left(\bar{E}{\bar{W}}^{*}\right)}_{\alpha }^{U}$	0.655	0.557	0.391	0.419	0.625	0.497	0.488	0.454
1	${\left(\bar{E}{\bar{W}}^{*}\right)}_{\alpha }^{L}$	0.671	0.574	0.406	0.434	0.641	0.511	0.503	0.468
	${\left(\bar{E}{\bar{W}}^{*}\right)}_{\alpha }^{U}$	0.671	0.574	0.406	0.434	0.641	0.511	0.503	0.468

**Table 13:** The importance ranking of engineering property indexes.

	*EP*_1_	*EP*_2_	*EP*_3_	*EP*_4_	*EP*_5_	*EP*_6_	*EP*_7_	*EP*_8_
*MC*	0.241	0.295	0.263	0.259	0.344	0.21	0.329	0.225
Ranking	6	3	4	5	1	8	2	7
*DT*	0.412	0.345	0.237	0.259	0.393	0.304	0.302	0.277
Ranking	1	3	8	7	2	4	5	6
*MT*	0.35	0.236	0.293	0.198	0.334	0.216	0.253	0.258
Ranking	1	6	3	8	2	7	5	4

Because the market customer group is limited by the production background and process knowledge, it is impossible to effectively evaluate the correlation between the technical property indexes and the engineering property indexes. Therefore, in the second stage, we just collect the evaluation information of process design group (DT) and product manufacturing group (MT). According to the correlation matrix and the autocorrelation matrix, combing with Eqs (24) and (25). The importance mean of each index is calculated by mean method. we can obtain the importance ranking of technical property indexes of multi-agent, as shown in Table 14.

**Table 14:** The importance ranking of technical property indexes.

	*TP*_1_	*TP*_2_	*TP*_3_	*TP*_4_	*TP*_5_	*TP*_6_	*TP*_7_	*TP*_8_	*TP*_9_	*TP*_10_	*TP*_11_	*TP*_12_	*TP*_13_	*TP*_14_
*DT*	0.709	0.138	0.175	0.407	0.630	0.464	0.536	0.426	0.811	0.630	0.098	0.828	0.928	0.302
Ranking	4	13	12	10	5	8	7	9	3	6	14	2	1	11
*MT*	0.662	0.202	0.194	0.170	0.749	0.589	0.497	0.494	0.399	0.447	0.246	0.886	0.701	0.309
Ranking	4	12	13	14	2	5	6	7	9	8	11	1	3	10
*QT*(mean)	0.685	0.170	0.185	0.289	0.689	0.527	0.516	0.460	0.605	0.539	0.172	0.857	0.814	0.305
Ranking	4	14	12	11	3	7	8	9	5	6	13	1	2	10

**Fig 6:**
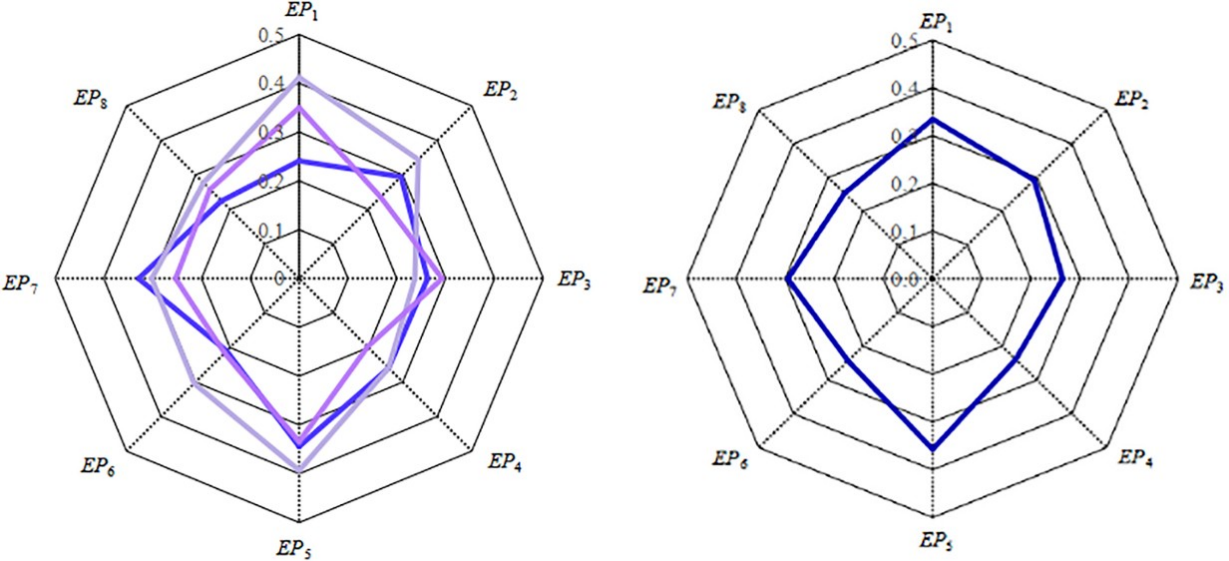
The importance of engineering property indexes. a. The importance of muti-agent. b. The importance means.

**Fig 7:**
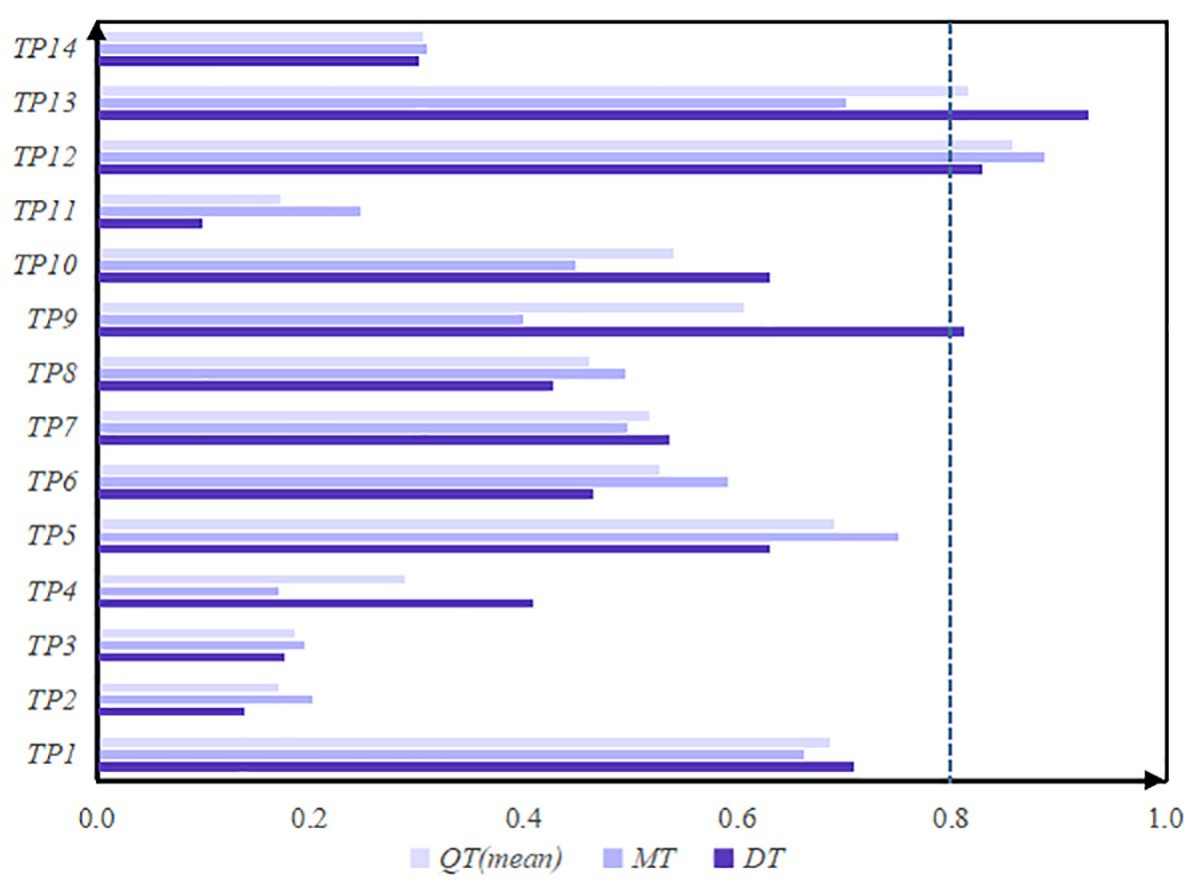
The importance of technical characteristics property indexes.

To further facilitate the study, Tables 13 and 14 are drawn in Figs 6 and 7.

Through the quantitative analysis of engineering property indexes, it is known that Gree should first improve or innovate the product operation efficiency (*EP*_5_) that multi-agent are most concerned about. Then the engineering property indexes most concerned by multi-agent ranked second and third are Motor units (*EP*_1_) and Control settings (*EP*_7_). Further quantitative analysis of the product’s property indexes shows that the importance of frequency conversion design (*TP*_12_), filter screen design (*TP*_13_), operating range design (*TP*_5_), air inlet/outlet design (*TP*_1_), low/high voltage protection design (*TP*_9_) rank in the top five of the overall technical indexes. In order to improve air purification capacity during the COVID-19 pandemic, these five aspects are given priority in the transformation or replacement of the KFR air-conditioning.

## 6. Conclusion

This paper studies the KFR air-conditioning innovation of Gree based on the multi-agent perspective under fuzzy environment. This paper proposes a product innovation index ranking method considering multi-agent demand preferences in a fuzzy environment. The engineering property indexes and the technical property indexes should be given priority in the next stage of product innovation.

Firstly, the Kano model is used to classify multi-agent demands. Secondly, QFD model is used to decompose multi-agent demand into engineering property indexes (product planning stage) and technical property indexes (process planning stage). Then, based on fuzzy sets, we can get the fuzzy evaluation information of the expert group (market customer group, process design group, product manufacturing group). Furthermore, the cut-set is used to transform the fuzzy evaluation information into accurate information. Finally, the model is used to analyze engineering and technical property indexes of the KFR air-conditioning which should be focused on in the next improvement or innovation. The model proposed in this paper fully considers the demand preferences and fuzzy environment of multi-agent for product innovation in the actual innovation process, and analyzes the two stages of product innovation.

The main contributions of this paper are in two aspects. On the one hand, the paper considers the impact of market demands and technological progress on product innovation, and uses fuzzy sets to collect multi-agent evaluation information to reduce the loss of evaluation information. On the other hand, through two-stage continuous decomposition, product demands are gradually decomposed into product planning designs and manufacturing process designs (that is, from product demands to product designs to process designs), which refines the product design process and makes the design tasks of the design department and the process department more clear. The method is clear and easy to operate, which lays a foundation for the research on critical factors of the same type of air-conditioning innovation.

Products that meet market demands is the fundamental goal of an enterprise’s production pursuit. Grasping market demands and technological innovation trends, adjusting product functions and structure, thereby extending product life cycle, this is a very worthwhile issue for enterprises to study. This paper focuses on determining the critical factors of air-conditioning innovation during the COVID-19 pandemic. The proposed methodology has demonstrated high flexibility and the way in which decision-making based on uncertain information can be improved. The method can be widely used in the innovation process of industrial and manufacturing products, but in specific applications, it is necessary to analyze specific issues. In addition, because the calculation of this paper is complex, in the future, we should explore how to use intelligent algorithms to solve the model.

## Supporting information

S1 File Minimal data set. (DOCX)
