# Supplementary material for: Determining the critical factors of air-conditioning innovation using an integrated model of fuzzy Kano-QFD during the COVID-19 pandemic: The perspective of air purification
Source: PLoS One. 2021 Jul 27;16(7):e0255051. doi: 10.1371/journal.pone.0255051 (PMC8315527; doi:10.1371/journal.pone.0255051)
Supplement: S1 File — (DOCX) [file pone.0255051.s001.docx]

**S1 File （****minimal data set）**

These data are the minimal data set of Example simulation underlying the study, which can be shared publicly. Other experimental data belong to Gree Electric Appliances, Inc.of Zhuhai, which have some restrictions, if required, contact the corresponding author to obtain them.

**Table 1 Demand classification and evaluation information**

|  | Demand indexes | M | O | A | I | R | Total | KC | $x_{i}$ | $x_{i}^{Ie}$ | $x_{i}^{Ae}$ |
| --- | --- | --- | --- | --- | --- | --- | --- | --- | --- | --- | --- |
| MC | *CR*1 | 45 | 33 | 22 | 14 | 36 | 150 | M | 0.008 | 0.192 | 0.452 |
|  | *CR*2 | 24 | 30 | 56 | 15 | 25 | 150 | A | 0.898 | 0.359 | 0.981 |
|  | *CR*3 | 10 | 34 | 48 | 35 | 23 | 150 | A | 0.164 | 0.157 | 0.350 |
|  | *CR*4 | 16 | 52 | 22 | 35 | 25 | 150 | O | 0.324 | 0.201 | 0.475 |
| DT | *CR*1 | 17 | 2 | 2 | 4 | 5 | 30 | M | 0.050 | 0.263 | 0.505 |
|  | *CR*2 | 5 | 3 | 13 | 6 | 3 | 30 | A | 0.351 | 0.382 | 0.678 |
|  | *CR*3 | 14 | 5 | 2 | 4 | 5 | 30 | M | 0.205 | 0.214 | 0.517 |
|  | *CR*4 | 11 | 1 | 9 | 2 | 7 | 30 | M | 0.259 | 0.252 | 0.765 |
| MT | *CR*1 | 8 | 2 | 1 | 4 | 5 | 20 | M | 0.275 | 0.377 | 0.764 |
|  | *CR*2 | 11 | 2 | 0 | 4 | 3 | 20 | M | 0.302 | 0.069 | 0.998 |
|  | *CR*3 | 8 | 4 | 3 | 2 | 3 | 20 | M | 0.143 | 0.199 | 0.965 |
|  | *CR*4 | 7 | 5 | 2 | 4 | 2 | 20 | M | 0.368 | 0.124 | 0.325 |

**Table 2 The importance of multi-agent demands**

|  | Demand indexes | 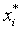 | *CS* | *DS* | *a_i_* | *b_i_* | *S_i_* | *SW_i_* |
| --- | --- | --- | --- | --- | --- | --- | --- | --- |
| MC | *CR*1 | 0.035 | 0.667 | 0.333 | 0.527 | 0.861 | 0.315 | 0.048 |
|  | *CR*2 | 0.867 | 0.733 | 0.267 | 0.272 | 0.005 | 0.651 | 0.288 |
|  | *CR*3 | 0.036 | 0.613 | 0.387 | 0.132 | -0.255 | -0.118 | -0.006 |
|  | *CR*4 | 0.449 | 0.600 | 0.400 | 0.200 | 0.400 | 0.490 | 0.463 |
| DT | *CR*1 | 0.050 | 0.700 | 0.300 | 0.633 | 0.933 | 0.268 | 0.053 |
|  | *CR*2 | 0.078 | 0.700 | 0.300 | 0.233 | -0.067 | 0.184 | 0.011 |
|  | *CR*3 | 0.300 | 0.700 | 0.300 | 0.633 | 0.933 | 0.078 | 0.091 |
|  | *CR*4 | 0.0136 | 0.700 | 0.300 | 0.633 | 0.933 | 0.291 | 0.010 |
| MT | *CR*1 | -0.264 | 0.550 | 0.450 | 0.158 | 0.608 | 0.487 | -0.336 |
|  | *CR*2 | 0.251 | 0.650 | 0.350 | 0.475 | 0.825 | 0.215 | 0.108 |
|  | *CR*3 | 0.010 | 0.750 | 0.250 | 0.791 | 1.041 | 0.242 | 0.005 |
|  | *CR*4 | 1 | 0.700 | 0.300 | 0.633 | 0.933 | -0.787 | -4.845 |

**Table 3 The evaluation information in the first stage (One export)**

|  | *EP*1 | *EP*2 | *EP*3 | *EP*4 | *EP*5 | *EP*6 | *EP*7 | *EP*8 |
| --- | --- | --- | --- | --- | --- | --- | --- | --- |
| *EP*1 | ● | ◎ | ▲ | ▲ | ● | ◎ | ◎ | ● |
| *EP*2 | ◎ | ● | ● | ▲ | ◎ | ◎ | ○ | ▲ |
| *EP*3 | ▲ | ● | ● | ● | ▲ | ▲ | ◎ | ▲ |
| *EP*4 | ▲ | ▲ | ● | ● | ○ | ▲ | ● | ▲ |
| *EP*5 | ● | ◎ | ▲ | ○ | ● | ● | △ | ◎ |
| *EP*6 | ◎ | ◎ | ▲ | ▲ | ● | ● | ▲ | △ |
| *EP*7 | ◎ | ○ | ◎ | ● | △ | ▲ | ● | ▲ |
| *EP*8 | ● | ▲ | ▲ | ▲ | ◎ | △ | ▲ | ● |
| *CR*1 | ● | ○ | ● | ▲ | △ | ◎ | ◎ | ● |
| *CR*2 | ◎ | ● | ○ | ▲ | ◎ | ● | ◎ | ◎ |
| *CR*3 | ● | ▲ | △ | ● | ● | ◎ | ● | ◎ |
| *CR*4 | ● | ▲ | ▲ | ▲ | ○ | ▲ | ▲ | ▲ |

**Table 4 The evaluation information in the second stage (One export)**

|  | *TP*1 | *TP*2 | *TP*3 | *TP*4 | *TP*5 | *TP*6 | *TP*7 | *TP*8 | *TP*9 | *TP*10 | *TP*11 | *TP*12 | *TP*13 | *TP*14 |
| --- | --- | --- | --- | --- | --- | --- | --- | --- | --- | --- | --- | --- | --- | --- |
| *TP*_1_ | ● | ▲ | ◎ | ● | ◎ | ◎ | △ | △ | △ | ▲ | ▲ | ○ | ○ | △ |
| *TP*_2_ | ▲ | ● | △ | ▲ | ▲ | ▲ | △ | ▲ | ▲ | ○ | △ | ▲ | ▲ | ▲ |
| *TP*_3_ | ◎ | △ | ● | ● | ● | ◎ | ◎ | ◎ | ◎ | ▲ | △ | ○ | ▲ | ▲ |
| *TP*_4_ | ● | ▲ | ● | ● | ● | ● | ○ | ○ | ○ | ▲ | ▲ | ◎ | ◎ | ▲ |
| *TP*_5_ | ◎ | ▲ | ● | ● | ● | ● | ▲ | ◎ | ◎ | ▲ | ▲ | ◎ | ▲ | ▲ |
| *TP*_6_ | ◎ | ▲ | ◎ | ● | ● | ● | ▲ | ● | ● | ▲ | ▲ | ○ | ▲ | ▲ |
| *TP*_7_ | △ | △ | ◎ | ○ | ▲ | ▲ | ● | ○ | ○ | ▲ | ▲ | ◎ | ▲ | ▲ |
| *TP*_8_ | △ | ▲ | ◎ | ○ | ◎ | ● | ○ | ● | ● | ▲ | ▲ | ◎ | ▲ | ▲ |
| *TP*_9_ | △ | ▲ | ◎ | ○ | ◎ | ● | ○ | ● | ● | ▲ | ▲ | ▲ | ◎ | ▲ |
| *TP*_10_ | ▲ | ○ | ▲ | ▲ | ▲ | ▲ | ▲ | ▲ | ▲ | ● | ◎ | ▲ | ▲ | ▲ |
| *TP*_11_ | ▲ | △ | △ | ▲ | ▲ | ▲ | ▲ | ▲ | ▲ | ◎ | ● | ▲ | ▲ | ▲ |
| *TP*_12_ | ○ | ▲ | ○ | ◎ | ◎ | ○ | ◎ | ◎ | ◎ | ▲ | ▲ | ● | ◎ | ◎ |
| *TP*_13_ | ○ | ▲ | ▲ | ◎ | ▲ | ▲ | ▲ | ▲ | ▲ | ▲ | ▲ | ◎ | ● | ◎ |
| *TP*_14_ | △ | ▲ | ▲ | ▲ | ▲ | ▲ | ▲ | ▲ | ▲ | ▲ | ▲ | ◎ | ◎ | ● |
| *EP*_1_ | ● | ● | ● | ● | ● | △ | △ | △ | △ | ▲ | ▲ | ○ | ○ | ○ |
| *EP*_2_ | ◎ | ○ | ○ | △ | △ | ● | ● | ● | ● | ▲ | ◎ | ▲ | ▲ | ▲ |
| *EP*_3_ | ▲ | ○ | ○ | ▲ | ○ | ○ | ○ | ○ | ◎ | ● | ▲ | ▲ | ▲ | ▲ |
| *EP*_4_ | ▲ | ▲ | ▲ | ▲ | ▲ | ▲ | ▲ | ▲ | ▲ | ● | ● | ▲ | ▲ | ▲ |
| *EP*_5_ | ◎ | ▲ | ▲ | ◎ | ○ | ○ | ○ | ○ | ○ | ▲ | ▲ | ● | ● | ◎ |
| *EP*_6_ | △ | ▲ | ▲ | ◎ | △ | ▲ | ▲ | △ | △ | ▲ | ▲ | ◎ | △ | ● |
| *EP*_7_ | ▲ | ▲ | ▲ | ▲ | ○ | ○ | ○ | ○ | ○ | ▲ | ◎ | ▲ | ▲ | ◎ |
| *EP*_8_ | ● | ▲ | ▲ | ● | ○ | ○ | ○ | ○ | ○ | ▲ | ▲ | ○ | ◎ | ▲ |

**Table 5 Fuzzy value of evaluation information in the first stage (DT)**

|  | *EP*_1_ | *EP*_2_ | *EP*_3_ | *EP*_4_ | *EP*_5_ | *EP*_6_ | *EP*_7_ |
| --- | --- | --- | --- | --- | --- | --- | --- |
| *EP*_1_ | (1.000,1.000,1.000) | (0.514,0.602,0.690) | (0.033,0.131,0.209) | (0.066,0.164,0.242) | (0.754,0.835,0.924) | (0.522,0.610,0.698) | (0.483,0.571,0.659) |
| *EP*_2_ | (0.514,0.602,0.690) | (1.000,1.000,1.000) | (0.718,0.799,0.888) | (0.035,0.133,0.211) | (0.582,0.670,0.758) | (0.438,0.526,0.614) | (0.372,0.482,0.592) |
| *EP*_3_ | (0.033,0.131,0.209) | (0.718,0.799,0.888) | (1.000,1.000,1.000) | (0.691,0.772,0.861) | (0.024,0.122,0.200) | (0.057,0.155,0.233) | (0.417,0.505,0.593) |
| *EP*_4_ | (0.066,0.164,0.242) | (0.035,0.133,0.211) | (0.691,0.772,0.861) | (1.000,1.000,1.000) | (0.409,0.519,0.629) | (0.051,0.149,0.227) | (0.765,0.846,0.935) |
| *EP*_5_ | (0.754,0.835,0.924) | (0.582,0.670,0.758) | (0.024,0.122,0.200) | (0.409,0.519,0.629) | (1.000,1.000,1.000) | (0.755,0.836,0.925) | (0.133,0.217,0.301) |
| *EP*_6_ | (0.522,0.610,0.698) | (0.438,0.526,0.614) | (0.057,0.155,0.233) | (0.051,0.149,0.227) | (0.755,0.836,0.925) | (1.000,1.000,1.000) | (0.150,0.248,0.326) |
| *EP*_7_ | (0.483,0.571,0.659) | (0.372,0.482,0.592) | (0.417,0.505,0.593) | (0.765,0.846,0.935) | (0.133,0.217,0.301) | (0.150,0.248,0.326) | (1.000,1.000,1.000) |
| *EP*_8_ | (0.775,0.856,0.945) | (0.135,0.233,0.311) | (0.076,0.174,0.252) | (0.038,0.136,0.214) | (0.468,0.556,0.644) | (0.111,0.195,0.279) | (0.034,0.132,0.210) |
| *CR*_1_ | (0.804,0.885,0.974) | (0.582,0.670,0.762) | (0.512,0.593,0.682) | (0.053,0.151,0.229) | (0.216,0.300,0.384) | (0.492,0.580,0.668) | (0.638,0.727,0.815) |
| *CR*_2_ | (0.689,0.777,0.865) | (0.582,0.670,0.763) | (0.515,0.625,0.735) | (0.094,0.192,0.270) | (0.543,0.631,0.719) | (0.752,0.833,0.922) | (0.532,0.620,0.708) |
| *CR*_3_ | (0.794,0.875,0.964) | (0.582,0.670,0.764) | (0.185,0.269,0.353) | (0.755,0.836,0.925) | (0.792,0.873,0.962) | (0.494,0.582,0.670) | (0.792,0.873,0.962) |
| *CR*_4_ | (0.773,0.854,0.943) | (0.582,0.670,0.765) | (0.088,0.186,0.264) | (0.120,0.218,0.296) | (0.342,0.452,0.562) | (0.046,0.144,0.222) | (0.054,0.152,0.230) |

**Table 6 The importance of engineering property indexes**

|  | *EW* | | | | | | | |
| --- | --- | --- | --- | --- | --- | --- | --- | --- |
|  | *EP*_1_ | *EP*_2_ | *EP*_3_ | *EP*_4_ | *EP*_5_ | *EP*_6_ | *EP*_7_ | *EP*_8_ |
| *MC* | (0.513,0.671,0.850) | (0.410,0.574,0.758) | (0.255,0.406,0.566) | (0.281,0.434,0.601) | (0.479,0.641,0.828) | (0.368,0.511,0.673) | (0.352,0.503,0.672) | (0.333,0.468,0.620) |
| *DT* | (0.543,0.709,0.898) | (0.428,0.602,0.796) | (0.269,0.428,0.597) | (0.303,0.465,0.640) | (0.507,0.678,0.874) | (0.387,0.538,0.708) | (0.377,0.535,0.713) | (0.353,0.495,0.655) |
| *MT* | (0.453,0.596,0.758) | (0.358,0.507,0.674) | (0.219,0.355,0.500) | (0.244,0.383,0.534) | (0.423,0.570,0.739) | (0.323,0.453,0.600) | (0.308,0.445,0.598) | (0.296,0.418,0.556) |
|  | *EW^*^* | | | | | | | |
| *MC* | (0.092,0.159,0.284) | (0.074,0.136,0.254) | (0.046,0.096,0.189) | (0.050,0.103,0.201) | (0.086,0.152,0.277) | (0.066,0.121,0.225) | (0.063,0.120,0.225) | (0.060,0.111,0.207) |
| *DT* | (0.092,0.159,0.284) | (0.073,0.135,0.251) | (0.046,0.096,0.189) | (0.052,0.104,0.202) | (0.086,0.152,0.276) | (0.066,0.121,0.224) | (0.064,0.120,0.225) | (0.060,0.111,0.207) |
| *MT* | (0.091,0.159,0.289) | (0.072,0.135,0.257) | (0.044,0.095,0.191) | (0.049,0.103,0.204) | (0.085,0.153,0.282) | (0.065,0.122,0.229) | (0.062,0.119,0.228) | (0.060,0.112,0.212) |

**Table 7 The importance under** $\boldsymbol{\alpha}$ **cut-set ((taking DT as an example)**

| $\alpha$ | $\left( \bar{E}\bar{W}^{*} \right)_{\alpha}$ | *EP*_1_ | *EP*_2_ | *EP*_3_ | *EP*_4_ | *EP*_5_ | *EP*_6_ | *EP*_7_ | *EP*_8_ |
| --- | --- | --- | --- | --- | --- | --- | --- | --- | --- |
| 0 | $\left( \bar{E}\bar{W}^{*} \right)_{\alpha}^{L}$ | 0.850 | 0.758 | 0.566 | 0.601 | 0.828 | 0.673 | 0.672 | 0.620 |
|  | $\left( \bar{E}\bar{W}^{*} \right)_{\alpha}^{U}$ | 0.513 | 0.410 | 0.255 | 0.281 | 0.479 | 0.368 | 0.352 | 0.333 |
| 0.1 | $\left( \bar{E}\bar{W}^{*} \right)_{\alpha}^{L}$ | 0.832 | 0.740 | 0.550 | 0.585 | 0.809 | 0.656 | 0.655 | 0.605 |
|  | $\left( \bar{E}\bar{W}^{*} \right)_{\alpha}^{U}$ | 0.529 | 0.426 | 0.270 | 0.296 | 0.495 | 0.382 | 0.367 | 0.347 |
| 0.2 | $\left( \bar{E}\bar{W}^{*} \right)_{\alpha}^{L}$ | 0.814 | 0.721 | 0.534 | 0.568 | 0.790 | 0.640 | 0.638 | 0.590 |
|  | $\left( \bar{E}\bar{W}^{*} \right)_{\alpha}^{U}$ | 0.545 | 0.443 | 0.285 | 0.311 | 0.511 | 0.396 | 0.382 | 0.360 |
| 0.3 | $\left( \bar{E}\bar{W}^{*} \right)_{\alpha}^{L}$ | 0.796 | 0.703 | 0.518 | 0.551 | 0.772 | 0.624 | 0.621 | 0.574 |
|  | $\left( \bar{E}\bar{W}^{*} \right)_{\alpha}^{U}$ | 0.561 | 0.459 | 0.300 | 0.327 | 0.528 | 0.411 | 0.397 | 0.374 |
| 0.4 | $\left( \bar{E}\bar{W}^{*} \right)_{\alpha}^{L}$ | 0.779 | 0.685 | 0.502 | 0.535 | 0.753 | 0.608 | 0.604 | 0.559 |
|  | $\left( \bar{E}\bar{W}^{*} \right)_{\alpha}^{U}$ | 0.576 | 0.475 | 0.315 | 0.342 | 0.544 | 0.425 | 0.412 | 0.387 |
| 0.5 | $\left( \bar{E}\bar{W}^{*} \right)_{\alpha}^{L}$ | 0.761 | 0.666 | 0.486 | 0.518 | 0.734 | 0.592 | 0.588 | 0.544 |
|  | $\left( \bar{E}\bar{W}^{*} \right)_{\alpha}^{U}$ | 0.592 | 0.492 | 0.330 | 0.357 | 0.560 | 0.439 | 0.427 | 0.401 |
| 0.6 | $\left( \bar{E}\bar{W}^{*} \right)_{\alpha}^{L}$ | 0.743 | 0.648 | 0.470 | 0.501 | 0.716 | 0.576 | 0.571 | 0.529 |
|  | $\left( \bar{E}\bar{W}^{*} \right)_{\alpha}^{U}$ | 0.860 | 0.762 | 0.568 | 0.607 | 0.836 | 0.677 | 0.679 | 0.626 |
| 0.7 | $\left( \bar{E}\bar{W}^{*} \right)_{\alpha}^{L}$ | 0.725 | 0.629 | 0.454 | 0.484 | 0.697 | 0.559 | 0.554 | 0.514 |
|  | $\left( \bar{E}\bar{W}^{*} \right)_{\alpha}^{U}$ | 0.623 | 0.525 | 0.361 | 0.388 | 0.593 | 0.468 | 0.458 | 0.428 |
| 0.8 | $\left( \bar{E}\bar{W}^{*} \right)_{\alpha}^{L}$ | 0.707 | 0.611 | 0.438 | 0.468 | 0.678 | 0.543 | 0.537 | 0.498 |
|  | $\left( \bar{E}\bar{W}^{*} \right)_{\alpha}^{U}$ | 0.639 | 0.541 | 0.376 | 0.404 | 0.609 | 0.482 | 0.473 | 0.441 |
| 0.9 | $\left( \bar{E}\bar{W}^{*} \right)_{\alpha}^{L}$ | 0.689 | 0.592 | 0.422 | 0.451 | 0.660 | 0.527 | 0.520 | 0.483 |
|  | $\left( \bar{E}\bar{W}^{*} \right)_{\alpha}^{U}$ | 0.655 | 0.557 | 0.391 | 0.419 | 0.625 | 0.497 | 0.488 | 0.454 |
| 1 | $\left( \bar{E}\bar{W}^{*} \right)_{\alpha}^{L}$ | 0.671 | 0.574 | 0.406 | 0.434 | 0.641 | 0.511 | 0.503 | 0.468 |
|  | $\left( \bar{E}\bar{W}^{*} \right)_{\alpha}^{U}$ | 0.671 | 0.574 | 0.406 | 0.434 | 0.641 | 0.511 | 0.503 | 0.468 |

**Table 8 The importance ranking of engineering property indexes**

|  | *EP*_1_ | *EP*_2_ | *EP*_3_ | *EP*_4_ | *EP*_5_ | *EP*_6_ | *EP*_7_ | *EP*_8_ |
| --- | --- | --- | --- | --- | --- | --- | --- | --- |
| *MC* | 0.241 | 0.295 | 0.263 | 0.259 | 0.344 | 0.21 | 0.329 | 0.225 |
| Ranking | 6 | 3 | 4 | 5 | 1 | 8 | 2 | 7 |
| *DT* | 0.412 | 0.345 | 0.237 | 0.259 | 0.393 | 0.304 | 0.302 | 0.277 |
| Ranking | 1 | 3 | 8 | 7 | 2 | 4 | 5 | 6 |
| *MT* | 0.35 | 0.236 | 0.293 | 0.198 | 0.334 | 0.216 | 0.253 | 0.258 |
| Ranking | 1 | 6 | 3 | 8 | 2 | 7 | 5 | 4 |

**Table 9 The importance ranking of technical property indexes**

|  | *TP*1 | *TP*2 | *TP*3 | *TP*4 | *TP*5 | *TP*6 | *TP*7 | *TP*8 | *TP*9 | *TP*10 | *TP*11 | *TP*12 | *TP*13 | *TP*14 |
| --- | --- | --- | --- | --- | --- | --- | --- | --- | --- | --- | --- | --- | --- | --- |
| *DT* | 0.709 | 0.138 | 0.175 | 0.407 | 0.630 | 0.464 | 0.536 | 0.426 | 0.811 | 0.630 | 0.098 | 0.828 | 0.928 | 0.302 |
| Ranking | 4 | 13 | 12 | 10 | 5 | 8 | 7 | 9 | 3 | 6 | 14 | 2 | 1 | 11 |
| *MT* | 0.662 | 0.202 | 0.194 | 0.170 | 0.749 | 0.589 | 0.497 | 0.494 | 0.399 | 0.447 | 0.246 | 0.886 | 0.701 | 0.309 |
| Ranking | 4 | 12 | 13 | 14 | 2 | 5 | 6 | 7 | 9 | 8 | 11 | 1 | 3 | 10 |
| *QT*(mean) | 0.685 | 0.170 | 0.185 | 0.289 | 0.689 | 0.527 | 0.516 | 0.460 | 0.605 | 0.539 | 0.172 | 0.857 | 0.814 | 0.305 |
| Ranking | 4 | 14 | 12 | 11 | 3 | 7 | 8 | 9 | 5 | 6 | 13 | 1 | 2 | 10 |
